# The 2000HIV study: Design, multi-omics methods and participant characteristics

**DOI:** 10.3389/fimmu.2022.982746

**Published:** 2022-12-20

**Authors:** Wilhelm A. J. W. Vos, Albert L. Groenendijk, Marc J. T. Blaauw, Louise E. van Eekeren, Adriana Navas, Maartje C. P. Cleophas, Nadira Vadaq, Vasiliki Matzaraki, Jéssica C. dos Santos, Elise M. G. Meeder, Janeri Fröberg, Gert Weijers, Yue Zhang, Jingyuan Fu, Rob ter Horst, Christoph Bock, Rainer Knoll, Anna C. Aschenbrenner, Joachim Schultze, Linos Vanderkerckhove, Talent Hwandih, Elizabeth R. Wonderlich, Sai V. Vemula, Mike van der Kolk, Sterre C. P. de Vet, Willem L. Blok, Kees Brinkman, Casper Rokx, Arnt F. A. Schellekens, Quirijn de Mast, Leo A. B. Joosten, Marvin A. H. Berrevoets, Janneke E. Stalenhoef, Annelies Verbon, Jan van Lunzen, Mihai G. Netea, Andre J. A. M. van der Ven

**Affiliations:** ^1^ Department of Internal Medicine and Infectious Diseases, Radboudumc, Radboud University, Nijmegen, Netherlands; ^2^ Department of Internal Medicine and Infectious Diseases, OLVG, Amsterdam, Netherlands; ^3^ Department of Internal Medicine and Department of Medical Microbiology and Infectious diseases, Erasmus Medical Center (MC), Erasmus University, Rotterdam, Netherlands; ^4^ Department of Internal Medicine and Infectious Diseases, Elizabeth-Tweesteden Ziekenhuis, Tilburg, Netherlands; ^5^ Department of Psychiatry, Radboudumc, Radboud University, Nijmegen, Netherlands; ^6^ Donders Institute for Brain, Radboud University, Cognition and Behavior, Nijmegen, Netherlands; ^7^ Nijmegen Institute for Scientist-Practitioners in Addiction (NISPA), Radboud University, Nijmegen, Netherlands; ^8^ Medical UltraSound Imaging Center (MUSIC) Department of Medical Imaging, Radboudumc, Radboud University, Nijmegen, Netherlands; ^9^ Universitair Medisch Centrum Groningen, University of Groningen, Groningen, Netherlands; ^10^ Center for Molecular Medicine (CeMM) Research Center for Molecular Medicine of the Austrian Academy of Sciences, Vienna, Austria; ^11^ Medical University of Vienna, Center for Medical Statistics, Informatics and Intelligent Systems (CeMSIIS), Institute of Artificial Intelligence, Vienna, Austria; ^12^ Systems Medicine, Deutsches Zentrum für Neurodegenerative Erkrankungen (DZNE) eingetragener Verein (e.V.), Bonn, Germany; ^13^ Genomics & Immunoregulation, Life & Medical Sciences (LIMES) Institute, University of Bonn, Bonn, Germany; ^14^ Platform for Single Cell Genomics and Epigenomics (PRECISE), DZNE and University of Bonn, Bonn, Germany; ^15^ HIV Cure Research Center, Department of Internal Medicine and Pediatrics, Ghent University Hospital, Ghent University, Ghent, Belgium; ^16^ Medical Science Department, Sysmex Europe Societas Europaea (SE), Norderstedt, Germany; ^17^ Clinical Development, ViiV Healthcare, Durham, NC, United States; ^18^ Translational Medical Research, ViiV Healthcare, Brentford, United Kingdom; ^19^ Department of Medical Genetics, Iuliu Hatieganu University of Medicine and Pharmacy, Cluj-Napoca, Romania; ^20^ Department of Immunology and Metabolism, Life and Medical Sciences Institute, University of Bonn, Bonn, Germany

**Keywords:** multi-omics, HIV-1, non-AIDS comorbidities, cardiovascular disease, hepatic disease, COVID-19, HIV reservoir, HIV extreme phenotype

## Abstract

**Background:**

Even during long-term combination antiretroviral therapy (cART), people living with HIV (PLHIV) have a dysregulated immune system, characterized by persistent immune activation, accelerated immune ageing and increased risk of non-AIDS comorbidities. A multi-omics approach is applied to a large cohort of PLHIV to understand pathways underlying these dysregulations in order to identify new biomarkers and novel genetically validated therapeutic drugs targets.

**Methods:**

The 2000HIV study is a prospective longitudinal cohort study of PLHIV on cART. In addition, untreated HIV spontaneous controllers were recruited. In-depth multi-omics characterization will be performed, including genomics, epigenomics, transcriptomics, proteomics, metabolomics and metagenomics, functional immunological assays and extensive immunophenotyping. Furthermore, the latent viral reservoir will be assessed through cell associated HIV-1 RNA and DNA, and full-length individual proviral sequencing on a subset. Clinical measurements include an ECG, carotid intima-media thickness and plaque measurement, hepatic steatosis and fibrosis measurement as well as psychological symptoms and recreational drug questionnaires. Additionally, considering the developing pandemic, COVID-19 history and vaccination was recorded. Participants return for a two-year follow-up visit. The 2000HIV study consists of a discovery and validation cohort collected at separate sites to immediately validate any finding in an independent cohort.

**Results:**

Overall, 1895 PLHIV from four sites were included for analysis, 1559 in the discovery and 336 in the validation cohort. The study population was representative of a Western European HIV population, including 288 (15.2%) *cis*-women, 463 (24.4%) non-whites, and 1360 (71.8%) MSM (Men who have Sex with Men). Extreme phenotypes included 114 spontaneous controllers, 81 rapid progressors and 162 immunological non-responders. According to the Framingham score 321 (16.9%) had a cardiovascular risk of >20% in the next 10 years. COVID-19 infection was documented in 234 (12.3%) participants and 474 (25.0%) individuals had received a COVID-19 vaccine.

**Conclusion:**

The 2000HIV study established a cohort of 1895 PLHIV that employs multi-omics to discover new biological pathways and biomarkers to unravel non-AIDS comorbidities, extreme phenotypes and the latent viral reservoir that impact the health of PLHIV. The ultimate goal is to contribute to a more personalized approach to the best standard of care and a potential cure for PLHIV.

## 1 Introduction

Chronic HIV infection leads to a dysregulated immune system in the vast majority of people living with HIV (PLHIV), even when complete viral suppression is achieved by long-term combinational antiretroviral therapy (cART). The persistent immune activation, ongoing inflammation, and accelerated immune ageing are associated with an array of common non-AIDS-related diseases such as cardiovascular disease (CVD) and non-alcoholic fatty liver disease (NAFLD) (1). These alterations within the functioning of the immune system hinders effective immunity against infectious diseases as well as non-infectious diseases such as cancer. The latent HIV reservoir prevents eradicative and functional cure, so that PLHIV continue to suffer from increased rates of non-AIDS comorbidities and infections (2). Mechanisms behind these phenomena are poorly understood. The 2000HIV Human Functional Genomics Partnership Program (2000HIV study) assesses in great depth the factors underlying immune dysregulation by employing a multi-omics approach on a large cohort (n=1895) of PLHIV.

With the advances in the various *omics* technologies, our understanding of the human genome, transcriptome, proteome, metabolome and microbiome has vastly increased over recent years, although the integration of these data has lagged behind (3, 4). This hampers the understanding of these complex biological pathways and their specific roles in health and chronic diseases. To fill this knowledge gap, the Human Functional Genomics Project (HFGP) (www.humanfunctionalgenomics.org) was started in October 2009 by researchers at the Radboud University Medical Center (Radboudumc), the University Medical Center Groningen, and the Broad Institute at Harvard-MIT, later joined by investigators at the university of Bonn. The first cohorts of healthy Western-European ancestry individuals have yielded important new insights into underlying genetic, host and environmental factors influencing human cell populations and cytokine responses, the molecular effects of the microbiome on immune responses, and the effects of environmental and genetic factors on T and B cell immune traits (5–8). The power of functional genomics approaches has been subsequently recognized (9).

In the following years, various additional cohorts recruited within the HFGP provided important new insights. These cohorts included obese subjects at high risk of ischemic vascular disease (300-OB), individuals with diabetes mellitus (200DM), subjects with hyperuricemia and gout (750Gout), cohorts from rural and urban areas of Tanzania (300-Tanzania), individuals vaccinated with BCG (300-BCG) and a cohort of 200 PLHIV using cART (200HIV pilot study) (10–20). In the 200HIV pilot study, we were able to show, among other things, that monocytes of PLHIV exhibited a sustained proinflammatory immune phenotype with priming of the IL-1β pathway (15). Although the comparison between PLHIV and healthy controls has given new insights (15–17), a larger cohort is required to assess differences between specific clinical phenotypes within the PLHIV group itself. This paper describes the large-scale analyses in a new cohort of 1895 PLHIV using cART, the 2000HIV study. The focus of the 2000HIV study is to define pathways and biomarker panels for the following HIV research priorities (21, 22) in PLHIV using long-term cART:

1. Persistent inflammation and accelerated immune ageing in PLHIV;
2. Non-AIDS-related comorbidities (cardiovascular disease, non-alcoholic fatty liver disease, psychiatric impairment, metabolic disorders, non-AIDS-cancers);
3. Extreme HIV phenotypes (spontaneous controllers, non-responders, rapid progressors as defined in **Supplementary Table S1**);
4. HIV reservoir size and composition.

In conclusion, the 2000HIV study will strive to improve our understanding of the pathophysiology of HIV offering options for a more personalized approach to the best standard of care and potential cure strategies for PLHIV, focusing on integrating multi-omics and prioritizing genetically validated therapeutic drug targets.

## 2 Methods and analysis

### 2.1 Study design

**Figure 1:**
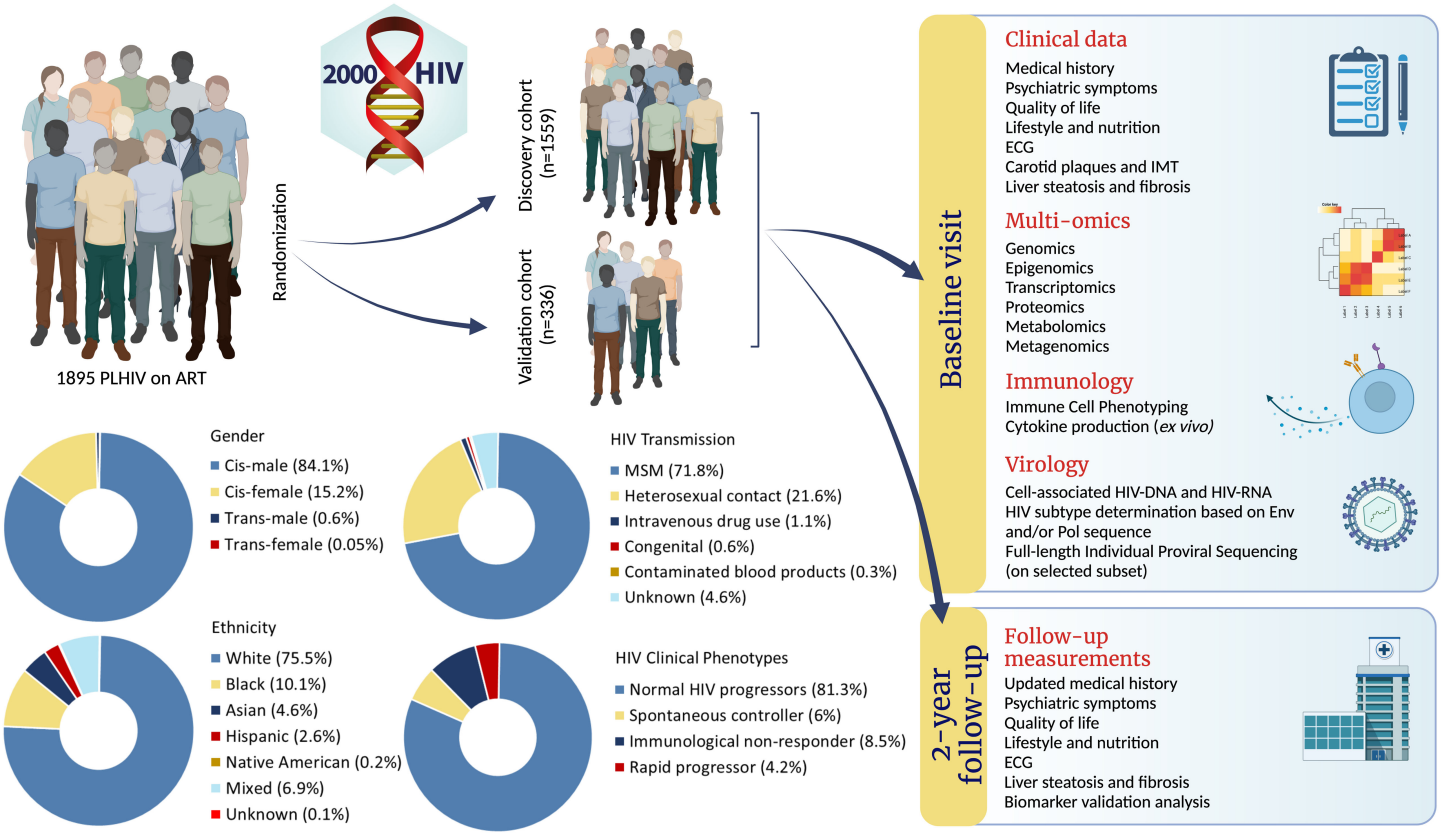
Abstract figure: Infographic of 2000HIV study. (Image created with BioRender.com).

The 2000HIV study is a prospective multicentric observational longitudinal cohort of PLHIV on stable cART (**Figure 1**). At baseline, participants’ clinical data were collected using questionnaires and patient medical records. Comorbidities (CVD and NAFLD) were assessed by imaging techniques (ultrasound, FibroScan^®^) and electrocardiogram (ECG). Whole blood was drawn for genetic, epigenomic, transcriptomic, proteomic, metabolomic, immunological, and virological analyses. Stool and saliva were collected for metagenomics. During a follow-up visit after two years, clinical data are again collected using questionnaires and patient medical records, NAFLD and CVD status are assessed using imaging techniques and ECGs. Blood samples are collected for biomarker and infection/inflammation parameter analysis. As of writing, baseline visits have been concluded and the two-year follow-up visits are ongoing.

### 2.2 Ethics approval and informed consent

The 2000HIV study protocol was approved by an accredited medical research ethics committee, the Independent Review Board Nijmegen (NL68056.091.81) and published at clinicaltrials.gov (ID: NCT03994835). Written informed consent was received from participants prior to inclusion in the study. All experiments with human samples were conducted according to the principles expressed in the Declaration of Helsinki.

### 2.3 Study population

Participants were recruited from October 2019 until October 2021. The 2000HIV study is made up of the discovery cohort and the validation cohort. Participants in the discovery cohort were recruited in three specialized Dutch HIV treatment centers, two university medical centers and one large general hospital (*Radboudumc Nijmegen, Erasmus MC Rotterdam, and OLVG Amsterdam*). Participants in the validation cohort were recruited in a separate medical center, a large general hospital (*Elisabeth-TweeSteden Ziekenhuis Tilburg*). Although the samples of the two subcohorts were collected separately, processing and measurements were identical. Inclusion criteria were: proven HIV-1 infection, age of 18 years or older, receiving cART for at least six months and with a latest HIV-1 RNA of less than 200 copies/mL. Additionally, individuals with spontaneous control of HIV-1 without cART could participate if viral loads had been lower than <10,000 copies/mL for at least five years, during which CD4^+^ T-cell counts were stable >500 cells/mm^3^. Exclusion criteria were: no informed consent, insufficient communication because of language barriers or other problems, current pregnancy, detectable viral hepatitis B or C DNA by polymerase chain reaction (PCR) or signs of any current acute infection. We aimed to enroll approximately 20% *cis*-women, 20% individuals with an ethnic ancestry from Sub-Sahara Africa, 2-3% spontaneous controllers, 3% immunologic non-responders, and 4-5% rapid progressors (see **Supplementary Table S1** for definitions).

### 2.4 Visit procedures

PLHIV were approached by their physician or HIV-nurse specialist and received both verbal and written information available in Dutch, English and French. At least 7 days later, individuals were contacted by the study team and asked whether they wanted to participate. If so, a baseline visit was planned for which the participants were asked to fast for 4 hours prior to the visit and not to brush their teeth for 12 hours. After signing informed consent, the in- and exclusion criteria were verified. The family and medical histories were obtained, and current medication was listed. During the study, the Coronavirus disease 2019 (COVID-19) pandemic emerged, therefore all COVID-19 related complaints, COVID-19 test results, hospital admissions and vaccinations were also recorded for participants that participated after the onset of the pandemic. Subsequently, liver steatosis and fibrosis were assessed using FibroScan^®^ (Echosens, Paris, France) and via 2D ultrasound using Computer Aided UltraSound (CAUS) for steatosis (23) and 2D Sound Touch Elastography (STE) for fibrosis (Mindray, Shenzhen, China). The latter equipment was also used to measure the intima-media thickness (IMT) and the presence of plaques in both common carotid arteries. Furthermore, in all participants a resting ECG was recorded and 90 mL of blood was drawn. Finally, after verbal and written instructions, urine and saliva samples were collected in sterile containers, those for saliva containing a special preservation buffer (DNA Genotek OM-501). After the visit, participants filled in a questionnaire (general demographics and health status, MATE-Q, nutritional intake, EQ5D-5L, HADS, BIS-11) digitally or on paper, available in Dutch, English and French. Participants were instructed (verbally and in writing) how to collect a stool sample at home, using a commercially available collection kit containing preservation buffer (DNA Genotek OM-200), which had to be sent to Radboudumc by mail.

The researchers obtained the complete medical history from the electronic patient records, using a structured questionnaire and stored the data in Castor EDC^®^ (Amsterdam, the Netherlands). Additionally, in the Netherlands, the HIV monitoring foundation (Stichting HIV Monitoring, SHM) has prospectively collected data of most PLHIV living in the Netherlands, known as the ATHENA cohort (24). For our study we used data from both hospital files and the ATHENA cohort. Data were compared and verified if needed. In case of discrepancy the medical history from the participant’s hospital was used.

At the second visit after two years, which is still ongoing until the mid of 2023, the medical history of the previous two years is evaluated using a structured questionnaire and data obtained from the electronic patient records. Furthermore, NAFLD is again assessed in a subgroup of participants using ultrasound imaging and FibroScan^®^. In all subjects, another 10mL blood is drawn and an additional ECG is recorded.

### 2.5 Documentation and measurements of non-AIDS related comorbidities

Patient records were screened for AIDS and non-AIDS clinical events, including malignancies, infections, hepatic illnesses, cardiovascular disease and neuropsychiatric comorbidities. Systematic assessment was performed for the following three non-AIDS related comorbidities: NAFLD, CVD and neuropsychiatric symptoms.

Hepatic steatosis and fibrosis were assessed with B-mode ultrasound and FibroScan^®^ both at baseline and during follow-up. To assess steatosis, the controlled attenuation parameter (CAP) in dB/m was measured using FibroScan^®^ and the residual attenuation coefficient (RAC) in dB/cm/MHz using computer-aided ultrasound (CAUS). To assess fibrosis, liver stiffness measurements in kPa were performed with transient elastography (FibroScan^®^) and shear-wave elastography with conventional ultrasound (**Supplementary Text 1**). The data of steatosis and fibrosis generated by the ultrasound were sent to Radboudumc and analyzed as described previously (23).

Cardiovascular risk profiles were constructed with the Framingham Risk Score, using data extracted from patient records (including blood pressure), lipids measured at baseline blood evaluation, and smoking history recorded through the questionnaire (25). The presence of subclinical cardiovascular diseases was assessed using B-mode ultrasound and data were sent to Radboudumc to score carotid artery intima-media thickness and presence and thickness of plaques (**Supplementary Text 2**). All cardiovascular clinical events were also recorded and both baseline and follow-up ECGs were sent to Erasmus MC, the Netherlands, where they were evaluated using Modular ECG Analysis System (MEANS) (**Supplementary Text 3**) (26, 27).

Previous psychiatric diagnoses were extracted from patient records and psychiatric symptoms and quality of life were evaluated by using self-report questionnaires. Symptoms of depression and anxiety were assessed through the Hospital Anxiety and Depression Scale (HADS) (28), symptoms of impulsivity through the Barratt Impulsiveness Scale (BIS-11) (29), and substance use through the Measurements in the Addictions for Triage and Evaluation (MATE-Q) (30). Quality of life was assessed through the EuroQol 5-Dimension 5-Level questionnaire (EQ-5D-5L) (31).

All participating centers used identical machines for ECG, ultrasound and FibroScan^®^ regarding brand, model, settings and calibration. Each clinical investigator followed the same training at the same time and had regular meetings with experts to ensure a standardized quality of imaging.

### 2.6 Samples processing

Biological body fluids were collected during daytime hours at the participating centers and sent overnight to the Radboudumc. The overnight shipping was at room temperature and samples were processed immediately in the morning the day after the day of baseline visit. The samples collected in the coordinating center, Radboudumc, were also stored overnight at room temperature to ensure identical handling across all centers. Upon arrival of the samples in the lab, whole blood obtained from EDTA tubes was used for hemocytometric analysis with the XN Sysmex haematology analyzer (Sysmex, Kobe), and for immunophenotyping using flow cytometry (CytoFLEX-LX, Beckman Coulter). Peripheral blood mononuclear cells (PBMC) were isolated using density gradient separation (Ficoll-paque) in SepMate™ tubes, as previously described (32). Whole blood was used for DNA isolation. Plasma (citrate and EDTA) and serum were aliquoted and stored at -80°C for future assessment.

### 2.7 Multi-omics measurements

In the 2000HIV study, all samples of a certain omics-layer are analyzed simultaneously. For each layer of *omics* data, strict quality control filters are applied (e.g., removing outlier samples), followed by proper pre-processing and normalization. Data of the various omics layers are integrated where appropriate. The different *omics* layers include genomics assessing single nuclear polymorphisms and whole genome sequencing in a subset of 200 participants, epigenomics assessing DNA methylation as well as open chromatin using the Assay for Transposase-Accesible Chromatin sequencing, transcriptomics in bulk PBMCs and in single cell (CD4, CD8, B-cells, Monocytes and NK cells) in a subset of 200 participants, and metagenomics on stool and saliva samples. In addition, plasma is used to for targeted proteomics and untargeted metabolomics. Also, immune cell functionality is evaluated through hemocytometric immune cell differentiation, extensive flowcytometry in three panels containing 17-20 markers each and ex-vivo PBMC 24 hours and 7 days cytokine production capacity after exposure to various stimuli. Furthermore, the HIV viral reservoir is evaluated through measurement of HIV-DNA and HIV RNA in all individuals and analysis of the proviral landscape in spontaneous controllers. Lastly, serology for COVID-19 and CMV infection is measured in plasma. Elaborate description of all methods can be found in in **Supplementary Text 4**. Multi-omics analyses are still ongoing at the time of writing.

#### 2.7.1 Hemocytometric immune cell differentiation

Whole blood was extensively analyzed with hemocytometry using the Sysmex XN series hematology analyzer (XN) (Sysmex, Kobe, Japan). We evaluated cell counts, cell complexity and cell reactivity, as described elsewhere (33). In brief, Sysmex uses an extensively validated flowcytometry approach with nucleic acid staining. Complexity (side scatter, SSC, X-axis, X), reactivity (fluorescence, SFL, Y-axis, Y) and size (forward scatter, FSC, Z-axis, Z) were measured for neutrophils, monocytes and lymphocytes. Intra-individual variability of these white blood cell sub-populations is indicated as width (WX, WY, WZ). We compared the 2000HIV study to the Dutch Lifelines cohort, which consists of 15,803 healthy individuals (33). Since sample to processing time affects red blood cell parameters (34), these were excluded. Neutrophils, lymphocytes and monocytes are significantly less affected by sample-to-processing time up to at least 72 hours (**Supplementary Figure S1**) (34). Comparisons were corrected for age, sex, BMI and smoking. Differences were considered significant and relevant, if the adjusted P-value was <0.05 and the Cohen’s d effect size was > 0.5. Additionally, we compared hemocytometry of the 2000HIV study to values observed in individuals during viral infections, bacterial infections, Dengue, Malaria, and COVID-19 (35–37).

Extensive characterization of these cohorts can be found elsewhere (35–37). In brief, peripheral blood of people presenting at the emergency room with fever was analyzed on XN and, subsequently, these individuals underwent extensive diagnostic testing to determine the causative pathogen as described elsewhere (35–37).

#### 2.7.2 CMV-serology

In all participants, serology for CMV (cytomegalovirus), herpes simplex virus 1 and 2 is measured on baseline samples. The CMV ELISA is performed on serum samples according to manufacturer’s protocol (GenWay, San Diego, California, US).

#### 2.7.3 COVID-19 serology

Serum Severe Acute Respiratory Syndrome Coronavirus 2 (SARS-CoV-2) anti-Spike (S) and anti-Nucleocapsid (N) IgG serology are retrospectively measured at baseline and after two-year follow up with a validated fluorescent-bead-based multiplex immunoassay, acquired on the Luminex FlexMap3D System, and used to discern past COVID-19 infection from uninfected individuals as described elsewhere (38). Detailed description of this method can be found in **Supplementary Text 5**. In addition, COVID-19 serology using ELISA was done in several PLHIV at the local hospital as part of routine care.

### 2.8 Sample size and statistical analysis

Earlier studies in the HFGP provided invaluable insights on the impact of genetic, environmental, and microbiome factors that are responsible for the variability of immune responses in health and disease (5–7). The use of cohorts of healthy individuals ranging from only 250 to 500 individuals together with patient cohorts led to a broader understanding of human immune responses. In the context of HIV infection, a cohort of approximately 200 PLHIV within the HFGP have provided novel knowledge on the factors and pathways that characterize the interaction between immune responses, the genetic background and the microbiome in virologically suppressed HIV-infected individuals using a system biology approach in which multi-omics data are being used (15–17). Increasing the number of individuals to nearly 2000 (2000HIV study) significantly increases the statistical power to detect robust and true factors and pathways that contribute to a persistent immune activation and dysfunction in virologically suppressed PLHIV. Diverse *omics* data are generated to delineate biological processes with the aim to identify candidate biomarkers and/or pathways that correlate with particular non-AIDS defining comorbidities, or are associated with clinical phenotypes, such as spontaneous controllers, rapid progressors and immunological non-responders.

## 3 Results

### 3.1 Baseline characteristics

Between October 2019 and October 2021, a total of 1910 PLHIV participated in the 2000HIV study. The follow-up visits have subsequently started and are aimed to be completed by October 2023. After recruitment, a total of 15 individuals were excluded from analysis due to severely confounding diseases unknowingly present at the time of sampling (e.g. acute syphilis, metastatic cancer). Therefore, 1895 participants met the inclusion criteria and were eligible for analysis. **Table 1** shows the baseline characteristics of the entire study and separately for the discovery and validation cohort (1559 and 336 participants, respectively). Notably, 112 individuals had also previously participated in the 200HIV pilot study between 2016-2018. As there is some overlap in analyses between 200HIV pilot study and the 2000HIV study, this will result in this subgroup with an actual median follow-up duration of 6.0 years (IQR 5.6 – 6.7 years) after the second visit for the 2000HIV study.

**Table 1:** Baseline characteristics of the study participants.

	Overall	Discovery cohort	Validation cohort
Number of participants	1910		
Included in analyses	1895	1559 (82.3%)	336 (17.7%)
Age in years, median (IQR)	53 (44-60)	53 (43-59)	53 (47-60)
Gender, N (%)			
*Cis-*male	1594 (84.1%)	1310 (84.0%)	284 (84.5%)
*Cis-female*	288 (15.2%)	236 (15.2%)	52 (15.5%)
*Trans-*male	1 (0.05%)	1 (0.06%)	0 (0%)
*Trans-*female	12 (0.6%)	12 (0.8%)	0 (0%)
Ethnical ancestry, N (%)			
White	1430 (75.5%)	1140 (73.1%)	290 (86.3%)
Black	192 (10.1%)	167 (10.7%)	25 (7.4%)
Asian	87 (4.6%)	76 (4.9%)	11 (3.3%)
Hispanic	50 (2.6%)	48 (3.1%)	2 (0.6%)
Native American	3 (0.2%)	1 (0.06%)	2 (0.6%)
Mixed*	131 (6.9%)	125 (8.0%)	6 (1.8%)
Unknown	2 (0.1%)	2 (0.1%)	0 (0%)
BMI (kg/m^2^), median (IQR)	25.0 (22.4-27.7)	24.9 (22.4-27.6)	25.5 (22.7-27.9)
Smoking status, N (%)			
Current smoker	559 (29.5%)	459 (29.4%)	100 (29.8%)
Previous smoker	571 (30.1%)	474 (30.4%)	97 (28.9%)
Never smoked	622 (32.8%)	516 (33.1%)	106 (31.5%)
Unknown	143 (7.5%)	110 (7.1%)	33 (9.8%)

*Mixed ethnicity is defined as having at least two grandparents of two separate ethnical ancestries.

BMI, Body Mass Index.

Overall, the majority of participants were *cis-*male (N=1594, 84.1%), and of white ethnical ancestry (N=1430, 75.5%). The major mode of transmission was MSM (Men who have Sex with Men; N=1360, 71.8%), as shown in **Table 2**. A total of 29.4% currently smokes whereas 30.1% has quit smoking. Median CD4 T^+^ cell nadir was 260 cells/mm^3^ (IQR 140-390) and median most recent CD4 T^+^ cell count was 700 (530–910) cells/mm^3^. Almost twenty-one percent of the individuals (N=395, 20.8%) had a history of an AIDS-defining illness. Time since HIV diagnosis ranged from six months to an exceptional 42 years (median 12.4 years, IQR 7.1-19.1 years) and time on cART between six months and 27 years (median 10.1 years, IQR 5.9-16.3 years). At baseline, 1868 individuals (98.6%) were on cART, some spontaneous controllers excepted. 288 individuals (15.2%) had a documented HIV seroconversion (incomplete immunoblot) or negative HIV test within six months prior to diagnosis. Of these, 88 (4.6%) started with cART within one month after diagnosis, classifying them as having early cART exposure.

**Table 2:** Baseline HIV-related characteristics of study participants.

	Overall	Discovery cohort	Validation cohort
Mode of transmission, N (%)			
MSM	1360 (71.8%)	1126 (72.2%)	234 (69.6%)
Heterosexual contact	410 (21.6%)	322 (20.7%)	88 (26.2%)
Intravenous drug use	20 (1.1%%)	19 (1.2%)	1 (0.3%)
Congenital	11 (0.6%)	9 (0.6%)	2 (0.6%)
Contaminated blood products	6 (0.3%)	6 (0.4%)	0 (0%)
Unknown/several possibilities	88 (4.6%)	77 (4.9%)	11 (3.3%)
Time since diagnosis in years, median (IQR)	12.4 (7.1-19.1)	13.0 (7.8-19.4)	10.2 (5.3-16.3)
CD4 nadir in cells/*mm* ^3^, median (IQR)	260 (140-390)	250 (140-390)	270 (130-410)
Most recent CD4 count in cells/*mm* ^3^, median (IQR)	700 (530-910)	712 (545-926)	660 (478-820)
Most recent CD8 count in cells/*mm* ^3^, median (IQR)	830 (610-1160)	830 (610-1160)	Not measured
Most recent CD4/CD8 ratio	0.88 (0.59-1.19)	0.88 (0.59-1.19)	Not measured
Viral load zenith in copies/ml, median (IQR)	100,000 (38,950 - 285,762)	100,000 (37,750-264,000)	559,970 (46,387-346,914)
People with viral load under quantification limit at most recent measurement, N (%)	1790 (94.5%)	1480 (94.9%)	310 (92.3%)
Any AIDS-defining diagnosis in previous medical history, N (%)	395 (20.8%)	323 (20.7%)	72 (21.4%)
History of AIDS-defining malignancy, N (%)	115 (6.1%)	97(6.2%)	18 (5.4%)
History of AIDS-defining infection, N (%)	256 (13.5%)	212 (13.6%)	44 (13.1%)
Other AIDS-defining diagnoses (Wasting, Progressive multifocal leukoencephalopathy, HIV encephalopathy), N (%)	167 (8.8%)	123 (7.9%)	44 (13.1%)
Time on cART in years, median (IQR)	10.1 (5.9-16.3)	10.5 (6.4-16.5)	7.7 (4.8-14.1)
One-drug ART regime in use, N (%)	1 (0.05%)	1 (0.06%)	0 (0%)
Two-drug cART regime in use, N (%)	267 (14.1%	234 (15.0%)	33 9.8%)
Three-drug cART regime in use, N (%)	1576 (83.2%)	1280 (82.1%)	296 (88.1%)
More-than-three-drug cART regime in use, N (%)	24 (1.3%)	18 (1.2%)	6 (1.8%)
ART classes in use, N (%)			
NRTI	1811 (95.6%)	1482(95.1%)	329 (97.9%)
NNRTI	742 (39.2%)	646 (41.4%)	96 (28.6%)
PI	180 (9.5%)	151 (9.7%)	29 (8.6%)
INSTI	1039 (54.8%)	811 (52.0%)	228 (67.9%)
Entry inhibitor	1 (0.05%)	0 (0%)	1 (0.3%)
Diagnosis during acute HIV infection, N (%)	288 (15.2%)	239 (15.3%)	49 (14.6%)
Early cART (i.e. cART within first month of diagnosis of recent HIV infection), N (%)	88 (4.6%)	67 (4.3%)	21 (6.3%)
Extreme HIV phenotypes, N (%)			
Spontaneous controller	114 (6.0%)	102 (6.5%)	12(3.6%)
Immunological non-responder	162 (8.5%)	142 (9.1%)	20 (6.0%)
Rapid progressor	81 (4.3%)	72 (4.6%)	10 (3.0%)

N, Number of Participants; IQR, Inter Quartile Range; MSM, Men who have sex with men; mm^3^, Cubic Millimeter; cART, Combination Antiretroviral Therapy; ART, Antiretroviral Therapy; NRTI, Nucleoside Reverse Transcriptase inhibitors; NNRTI, Non-Nucleoside Reverse Transcriptase Inhibitors; PI, Protease Inhibitors; INSTI, Integrase Inhibitors.

In our study, 53 participants (2.8%) are being successfully treated for a chronic hepatitis B virus (HBV) infection (undetectable HBV-DNA) and 634 participants (33.5%) had an anti-HBc-detectable serology (**Table 3**). A total of 831 (43.9%) individuals had been vaccinated for HBV. Positive hepatitis A virus (HAV) serology was present in 1108 participants (58.5%; either vaccinated or previous infection) and 172 (9.1%) had a history of hepatitis C virus (HCV) infection. Finally, the vast majority of the participants had detectable anti-CMV IgG antibodies (N=1770, 93.8%, **Supplementary Figure S2**).

**Table 3:** Baseline viral co-infections of study participants.

	Overall	Discovery cohort	Validation cohort
Positive HAV serology, N (%)	1108 (58.5%)	1096 (70.3%)	48 (14.2%)
Previous infection, N (%)	221 (11.7%)	204 (13.1%)	17 (5.1%)
Previous vaccination, N (%)	347 (18.3%)	317 (20.3%)	30 (8.9%)
HAV antibodies present, unknown if acquired through vaccination or infection, N (%)	540 (28.5%)	539 34.6%)	1 (0.3%)
Negative HAV serology, N (%)	318 (16.8%)	309 (19.8%)	9 (2.7%)
Unknown HAV serology, N (%)	469 (24.7%)	190 (12.2%)	279 (83.0%)
Positive HBV serology, N (%)	1518 (80.1%)	1296 (83.1%)	222 (66.1%)
Previous infection, N (%)	634 (33.5%)	537 (34.4%)	97 (28.9%)
Previous vaccination, N (%)	831 (43.9%)	715 (45.9%)	116 (34.5%)
Chronic ART-suppressed infection, N (%)	53 (2.8%)	44 (2.8%)	9 (2.7%)
Negative HBV serology, N (%)	260 (13.7%)	224 (14.4%)	36 (10.7%)
Unknown HBV serology, N (%)	117 (6.2%)	39 (2.5%)	78 (23.1%)
Known previous HCV infection, N (%)	172 (9.1%)	147 (9.4%)	25 (7.4%)
Serum available for CMV serology, N (%)	1887 (99.6%)	1553 (99.6%)	334 (99.4%)
Positive CMV IgG serology, N (%)	1770 (93.8%)	89 (5.7%)	28 (8.3%)
CMV IgG titer in IU/mL, median (IQR)	690 (400-904)	682 (391-900)	703 (439-941)

HAV, Hepatitis A Virus; HBV, Hepatitis B infection; HCV, Hepatitis C Virus; CMV, Cytomegalovirus; IgG, Immunoglobulin G; IU, International Units; mL, Milliliter; N, Number of Participants; IQR, Inter Quartile Range.

Fibroscan^®^ and ultrasound were used to assess the liver characteristics (**Table 4**). Hepatic steatosis and fibrosis were assessed by ultrasound in 1750 and 1780 participants, respectively. Fibroscan^®^ measurements were performed in 1096 participants. After removing unsuccessful measurements (n = 13, mostly due to obesity) and applying a filter for validity (interquartile range divided by the median < 0.3), 1000 valid steatosis and 1007 valid fibrosis measurements remained.

**Table 4:** Liver, vascular and psychiatric measurements at baseline inclusion.

	Overall	Discovery cohort	Validation cohort
*Hepatic steatosis and fibrosis measurements*			
No. of Fibroscan CAP	1000	709	291
No. of Fibroscan LSM	1007	725	282
No. Liver ultrasound RAC	1750	1430	320
No. Liver ultrasound LSM	1780	1454	326
*Cardiovascular disease measurements*			
No. of carotid ultrasound imaging	1796	1467	329
No. of ECG	1649	1322	327
*Psychiatric symptoms and substance use measurements*			
HADS (anxiety and depression)			
No. of completed questionnaires	1642	1362	280
Severe symptoms of anxiety and stress (≥ 16), N(%)	288 (17.5%)	240 (17.6%)	48 (17.1%)
BIS-11 (impulsivity)			
No. of completed questionnaires	1642	1362	280
Highly impulsive (≥ 72), N (%)	136 (8.3%)	114 (8.4%)	22 (7.9%)
MATE-Q (substance use in past 30 days)			
No. of completed questionnaires	1654	1373	281
Heavy alcohol use, N (%)	179 (10.8%)	151 (11.0%)	31 (11.0%)
Cannabis use, N (%)	218 (13.2%)	203 (14.8%)	22 (7.8%)
XTC use, N (%)	148 (8.9%)	121 (8.8%)	29 (10.3%)
EQ-5D-5L (quality of life)			
No. of completed questionnaires	1660	1379	281
Any problems with:			
Mobility, N (%)	316 (19.2%)	270 (19.6%)	46 (16.4%)
Self-care, N (%)	47 (2.8%)	36 (2.6%)	11 (3.9%)
Usual activity, N (%)	287 (17.4%)	233 (16.9%)	54 (19.2%)
Pain or discomfort, N (%)	729 (44.1%)	605 (43.9%)	124 (44.1%)
Anxiety or depression, N (%)	609 (36.7%)	513 (37.2%)	96 (34.2%)
EQ-VAS, median (IQR)	80 (61-99)	80 (61-99)	80 (60-100)

N, Number of Participants; IQR, Inter Quartile Range; No., Number of participants; CAP, Controlled Attenuation Parameter; LSM, Liver Stiffness Measure; RAC, Residual Attenuation Coefficient; ECG, Electrocardiogram; HADS, Hospital Anxiety and Depression Scale; BIS-11, Barratt Impulsiveness Scale; MATE-Q, Measurements in the Addictions for Triage and Evaluation; EQ-5D-5L, 5-level EuroQol 5-dimensional questionnaire; EQ-VAS, EuroQol-visual analogue scales.

Carotid artery intima-media thickness was measured in 1796 participants, and 1649 ECGs were eligible for digital analyzation. Framingham risk scores could be calculated in 1365 participants, the remainder being too young or having incomplete data (**Table 4**). A high risk score of >20% 10-year risk of cardiovascular disease was found in 321 (16.9%) of the participants. In the whole study, 75 (4.0%) participants had a history of a myocardial infarction and 61 (3.2%) of an ischemic stroke (**Table 5**). The general questionnaire response rate was 88.9%. Blood was successfully collected from 1890 participants (99.7%) and saliva was obtained from all 1895 individuals. After participation, 1713 participants (90.4%) sent in their stools collected at home.

**Table 5:** Baseline previously recorded comorbidities in study participants.

	Overall	Discovery cohort	Validation cohort
Use of lipid lowering drugs, N (%)	391 (20.6%)	312 (20.0%)	79 (23.5%)
Hypertension, N (%)	474 (25.0%)	387 (24.8%)	87 (25.9%)
Myocardial infarction, N (%)	75 (4.0%)	58 (3.7%)	17 (5.1%)
Stroke, N (%)	61 (3.2%)	47 (3.0%)	14 (4.2%)
Type 1 diabetes, N (%)	4 (0.2%)	3 (0.2%)	1 (0.3%)
Type 2 diabetes, N (%)	90 (4.7%)	72 (4.6%)	18 (5.4%)
Hypothyroidism, N (%)	26 (1.4%)	22 (1.4%)	4 (1.2%)
Hyperthyroidism, N (%)	9 (0.5%)	8 (0.5%)	1 (0.3%)
COPD, N (%)	61 (3.2%)	51 (3.3%)	10 (3.0%)
Asthma, N (%)	101 (5.3%)	77 (4.9%)	24 (7.1%)
Liver steatosis, N (%)	204 (10.8%)	195 (12.5%)	9 (2.7%)
Liver cirrhosis, N (%)	16 (0.8%)	13 (0.8%)	3 (0.9%)
Alzheimers, N (%)	1 (0.05%)	1 (0.06%)	0 (0%)
Parkinsons, N (%)	3 (0.2%)	0	3 (0.9%)
Epilepsy, N (%)	29 (1.5%)	26 (1.7%)	3 (0.9%)
Depression, N (%)	311 (16.4%)	265 (17.0%)	46 (13.7%)
Anxiety disorder, N (%)	68 (3.6%)	64 (4.1%)	4 (1.2%)
Osteoporosis, N (%)	61 (3.2%)	51 (3.3%)	10 (3.0%)
Chronic renal failure according to Estimated Glomerular Filtration Rate (CDK-EPI) in ml/min/1,73 m^2^, N (%)			
Stadium 1: eGFR ≥ 90	712 (37.6%)	594 (38.1%)	118 (35.1%)
Stadium 2: eGFR 60-89	987 (52.1%)	812 (52.1%)	175 (52.1%)
Stadium 3: eGFR 30-59	183 (9.7%)	141 (9.0%)	42 (12.5%)
Stadium 4: eGFR 16-29	4 (0.2%)	3 (0.2%)	1 (0.3%)
Stadium 5: eGFR ≤ 15	3 (0.2%)	3 (0.2%)	0 (0%)
eGFR Unknown	6 (0.3%)	6 (0.4%)	0 (0%)
**Risk scores**			
No. Framingham score available, N (%)	1365 (72.0%)	1131 (72.5%)	234 (69.6%)
High Risk >20%	321 (16.9%)	266 (17.1%)	55 (16.3%)
Intermediate risk 10%-20%	485 (25.6%)	391 (25.1%)	94 (27.8%)
Low Risk <10%	559 (28.2%)	474 (30.4%)	85 (36.3%)
Unable to calculate/unknown	530 (28.0%)	428 (27.5%)	102 (30.4%)

N, Number of Participants; IQR, Inter Quartile Range; COPD, Chronic Obstructive Pulmonary Disease.

To provide a comprehensive overview, we report outcomes of completely filled out questionnaires only. Questionnaires on anxiety and depression (HADS) and impulsivity (BIS-11) were completed by 1642 participants (**Table 5**). Among these participants, 288 (17.5%) suffered from severe symptoms of anxiety and depression (total HADS score ≥ 16 points). In addition, 136 participants (8.3%) scored high on the impulsivity questionnaire (total BIS-11 score ≥ 72). Furthermore, evaluation of substance use by the MATE-Q in 1654 completed questionnaires showed that 179 (10.8%) were considered to be a heavy drinker. Also, 218 (13.2%) of the participants used cannabis and 148 (8.9%) XTC during the past 30 days. Overall, 1660 participants completed the questionnaire regarding quality of life (EQ-5D-5L), with a median EQ-VAS of 80 (IQR 61-99). Here, 729 participants (44.1%) reported problems related to pain, 609 (36.7%) reported problems related to anxiety and depression, and 316 (19.2%) reported mobility problems.

In our study, several extreme HIV-related phenotypes were included (**Table 2** and **Supplementary Table S1**). In total, 114 spontaneous controllers (34 elite and 80 viremic) were included, of whom 55 were transient controllers (48%; 10 elite and 45 viremic) who had lost spontaneous control before participation. In addition, 162 immunological non-responders and 81 rapid progressors were recruited.

### 3.2 COVID-19 incidence and vaccination among participants

On 27 February 2020, the first COVID-19 patient was diagnosed in the Netherlands and COVID-19 vaccination was distributed from January 2021 prioritizing health care workers and the elderly. Although PLHIV were officially prioritized from March 2021 onward, most received vaccination according to their age group. Due to a national lockdown, study enrolment was suspended between March 2020 and June 2020. In June 2020, study enrolment was resumed and was not suspended until the end of the baseline inclusion period in October 2021. The 381 participants (20.1%) that were enrolled before the lockdown of March-June 2020 are considered as not exposed to COVID-19, in contrast to the 1514 participants (79.9%) included after June 2020.

At baseline inclusion, 1040 of the 1514 (68.7%) at risk participants were unvaccinated, 213 (11.2% of total study) were partially vaccinated, 259 (13.7% of total study) were fully vaccinated and two (0.1% of total study) received at least one vaccination for COVID-19 but how often and when is unknown for these two (**Table 6A**). Full vaccination was considered 14 days or more after two doses of the Pfizer (BNT162b), Moderna (mRNA-1273), or AstraZeneca (ChAdOx1/Vaxzevria) vaccines, or 14 days or more after a single Johnson & Johnson (Ad26.COV2.S) vaccine. Two out of 259 fully vaccinated participants did not have S-serology seroconversion. The average time since the most recent vaccination was 45 days (IQR 17-66 days), and 94 (4.9%) participants received a COVID-19 vaccine less than 14 days prior to inclusion. None of the participants had received a third dose of the vaccine as part of the primary vaccination scheme, and booster rollout started only after baseline enrollment.

**Table 6:** COVID-19 status and serology measurement at baseline visit.

	Overall	Discovery cohort	Validation cohort
**A. Status COVID-19 vaccination at baseline inclusion**			
Fully vaccinated, N (%)	259 (13.7%)	237 (15.2%)	22 (6.5%)
Positive S-serology measured at baseline visit	257 (13.6%)	235 (99.2%)	22 (100%)
Negative S-serology measured at baseline visit	2 (0.1%)	2 (0.8%)	0 (0%)
Vaccinated but not fully, N (%)	213 (11.2%)	161 (10.3%)	52 (15.5%)
Vaccinated, but unknown how often or when, N (%)	2 (0.1%)	2 (0.1%)	0 (0%)
Unvaccinated, N (%)	1421 (75.0%)	1159 (74.3%)	262 (80.0%)
**B. Previous COVID-19 infection at baseline inclusion**			
Participant not exposed to COVID-19 (enrolled March 2020 or earlier), N (%)	381 (20.1%)	293 (18.8%)	88 (26.2%)
Participants potentially exposed to COVID-19 (enrolled June 2020 or later), N (%)	1514 (79.9%)	1266 (81.2%)	248 (73.8%)
Of participants exposed to COVID-19:			
At least one vaccination, N (%)	474 (31.3%)	400 (31.6%)	74 (29.8%)
Evidence of past COVID-19 infection, N (%)	55 (11.6%)	47 (11.8%)	8 (10.8%)
Reported previous positive PCR, N	37	30	7
Previous positive S-serology in own hospital before vaccination, N	21	20	1
No vaccination, N (%)	1040 (68.7%)	866 (68.4%)	174 (70.2%)		
Evidence of past COVID-19 infection, N (%)	179 (17.2%)	143 (16.5%)	36 (20.7%)
Previous reported positive PCR, N	40	32	8
Previous positive S-serology in own hospital before vaccination, N	30	30	0
Positive S-serology at baseline visit, N	175	141	34
			

S, anti-Spike protein; N, Number of Participants. COVID-19, Coronavirus Disease 2019; PCR, Polymerase Chain Reaction.

Evidence of previous COVID-19 infection was found in 234 (15.5%) of all 1514 participants as risk (included June 2020 or later) (**Table 6B**). Three of these participants had previously been admitted to the hospital due to COVID-19 of whom one was transferred to the ICU. Out of 1040 unvaccinated participants at risk of COVID-19, we identified in 179 (17.2%) participants a past COVID-19 infection (positive S-serology using Luminex at baseline identified 175 individuals, **Supplementary Figure S3**, 40 had reported a positive PCR during their visit and 30 had a positive S-serology at their local hospital as part of routine care at some time before baseline visit; 58 participants had overlapping positive tests). Among the 474 participants at risk of COVID-19 with at least one vaccination, we identified 55 (11.6%) with a past COVID-19 infection (37 participants reported a positive PCR and 21 had positive S-serology at their local hospital as part of routine care at some time before vaccination and before baseline visit; three had both tests positive). N-serology proved insufficient to diagnose a past COVID-19 infection in vaccinated people (**Supplementary Text 6**).

### 3.3 Neutrophils, monocytes and lymphocytes of PLHIV are distinct from people without HIV in hemocytometry Sysmex

**Figure 2:**
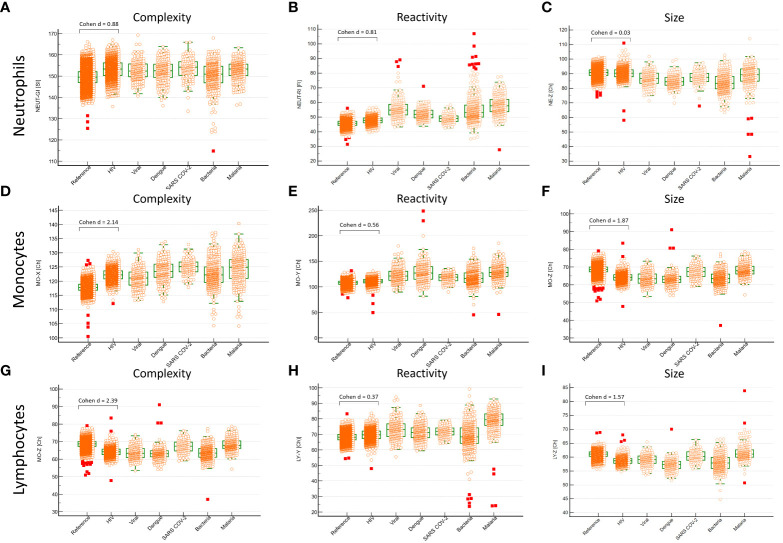
Neutrophils and monocytes of PLHIV have increased complexity and reactivity compared with people without HIV in Sysmex hemocytometry. N = 1895 PLHIV with suppressed viral loads were compared to N = 15,803 people without HIV. To place treated HIV in a context, PLHIV were displayed next to acute infectious disease cohorts. These other cohorts consisted of patients presenting with acute febrile disease at the ER who subsequently underwent extensive diagnostic testing. Cohen’s d is a measure of the effect size. Differences were considered significant if P < 0.05 and relevant if Cohen d effect size was > 0.5 **(A-C)** Neutrophils of PLHIV were higher in complexity and reactivity than people without HIV. **(D-F)** Monocytes of PLHIV were higher in complexity and reactivity but lower in size. **(G-I)** Lymphocytes of PLHIV were greater in complexity, smaller in size and similar in reactivity.

**Figure 3:**
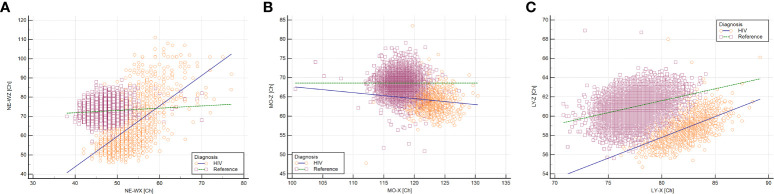
Neutrophils, monocytes and lymphocytes of PLHIV are distinct from people without HIV in Sysmex hemocytometry. Based on selected white blood cell characteristics, N = 1895 PLHIV (orange circles) with suppressed viral loads could easily be distinguished from N = 15,803 people without HIV (purple squares). **(A)** Neutrophils of PLHIV were unique in terms of intra-individual variability in both size and complexity. **(B)** Monocytes of PLHIV were distinct in size and complexity. **(C)** Lymphocytes of PLHIV were different in size and complexity.

We compared subsets of circulating immune cells as measured by new generation hematology analyzers in the 1895 PLHIV of the 2000HIV study and a reference population of 15,803 healthy adults. Circulating neutrophils, monocytes and lymphocytes were similar in cell counts (data not shown), yet differed in most other aspects (**Figures 2A–C**). For neutrophils, both granularity (NEUT-GI) and reactivity (NEUT-RI) were increased in PLHIV compared to healthy controls (Cohen’s d effect size = 0.89 and 0.81, respectively, adjusted P <0.001 for both). In fact, neutrophil granularity (NEUT-GI) of PLHIV showed similarities to the acute infectious disease cohorts. Intra-individual variability in neutrophil granularity (NE-WX) and reactivity (NE-WY) were increased in PLHIV compared to the healthy control cohort, with NE-WX being even higher than in other infection cohorts (**Supplementary Figures S4A-C**). Neutrophil size (NE-Z) was not distinct in PLHIV (Cohen’s d = 0.03). Considering only the intra-individual neutrophil variability in complexity (NE-WX) and size (NE-WZ), PLHIV could clearly be identified as separate from healthy controls (**Figure 3A**).

Monocytes in PLHIV were characterized by increased complexity (MO-X) and reactivity (MO-Y) (Cohen’s d = 2.14 and 0.56 respectively, adjusted P < 0.001 for both), while monocyte size (MO-Z) and intra-individual size variability (MO-WZ) were decreased (Cohen’s d = 1.87 and 1.37 respectively, adjusted P < 0.001 for both) (**Figures 2D-F**; **Supplementary Figures S4D–F**). Although the monocyte reactivity in PLHIV was not as high as found in other acute infectious diseases, the overall trend of a combination of increased monocyte reactivity and complexity with decreased size found in other infections was also found in PLHIV. Based on monocyte complexity (MO-X) and size (MO-Z), PLHIV were clearly distinct from people without HIV (**Figure 3B**).

Lymphocyte reactivity (LY-Y) did not substantially differ between PLHIV and healthy controls (Cohen’s d = 0.37). Nonetheless, lymphocyte complexity (LY-X) was higher in PLHIV, whereas lymphocyte size (LY-Z) was lower (Cohen’s d = 2.39 and 1.57, respectively, adjusted P < 0.001 for both) (**Figures 2G–I**). Intra-individual lymphocyte variability in complexity (LY-WX) and size (LY-WZ) were also substantially decreased in PLHIV (Cohen’s d = 1.48 and 3.42, respectively, adjusted P < 0.001 for both). Whereas the lymphocyte complexity and its variability (LY-X and LY-WX) in PLHIV were similar to an acute SARS-COV-2 infection, the decreased lymphocyte size and its variability (LY-Z and LY-WZ) in PLHIV were also observed in other acute viral and bacterial infections (**Figures 2G-I** and **Supplementary Figures S4G–I**). Lymphocyte complexity (LY-X) and size (LY-Z) clearly differentiated PLHIV from people without HIV (**Figure 3C**). Importantly, these HIV-related changes were not consistent with sample aging (**Supplementary Figure S1**)

PLHIV had high within-group variability in neutrophil, monocyte and lymphocyte markers (**Figure 2**). Of interest, within-group analysis of PLHIV revealed that observed changes were not driven by HIV-specific characteristics. Only small differences were found when we stratified the hemocytometry parameters by measures of HIV disease severity, such as CD4^+^ and CD8^+^ T cell count, CD4^+^ nadir, zenith viral load and extreme clinical phenotypes, including elite controllers (**Supplementary Table S3**).

## 4 Discussion

Here we described participant characteristics and the methodologies planned to perform multi-omics analyses of the 2000HIV study comprising 1895 PLHIV, of whom 112 also participated in a pilot study with a similar design between 2016-2018 (15). To our knowledge this is the largest study of PLHIV to date that analyzes multiple layers of *omics* simultaneously in order to comprehensively identify environmental, host genetic and non-genetic pathways and mechanisms that impact HIV-related comorbidities and the size of the viral reservoir. Due to careful data collection of most PLHIV in the Netherlands in the ATHENA cohort (24), which includes 21,155 of the estimated 24,000 PLHIV in the Netherlands, we were able to obtain a full overview of the general and HIV-related medical history of our participants.

Similar to the ATHENA cohort, the 2000HIV participants consisted mostly of middle aged MSM (39). Median history of HIV infection was 12.4 years. Median time on cART was 10.1 years while the median latest CD4 T-cell count was 700 cells/*mm* ^3^, indicating stable long-term suppressed HIV infection. Current (29.4%) and previous (30.1%) smoking was high in this study, as is found in most western countries’ HIV populations. Only 15.2% cis-females and 17.5% non-white ethnical ancestry participants were included, while the aim was to enroll 20% *cis-*females and 20% people of non-white ethnical ancestry. Our aim was not reached despite the employment of extra female research assistants to facilitate inclusion of these groups. However, the median percentage of women in HIV cure trials in the literature is reported to be even lower (11.1%) (40), and has been attributed to stigma, unfamiliarity with research, language barriers and practical challenges (41, 42). Nonetheless, the percentage of cis-females in our study is representative of PLHIV in the Netherlands (18.5%) (24, 39). Ethnical ancestry is not reported in the national registry (24, 39).

The study of specific clinical HIV-phenotypes will help in understanding the biology of HIV infection. Therefore, 114 spontaneous controllers, both transient and persistent, were included, as well as 81 rapid progressors and 162 immunological non-responders. Furthermore, 288 (15.2%) of participants included were initially diagnosed during the acute stage of HIV infection, as they presented with an incomplete immunoblot or a recent negative HIV test. As a reference, 13% of PLHIV diagnosed in the Netherlands between 2018-2021 had a negative HIV within six months prior, and this is 19% for MSM (39). Since the date of cART initiation is known in all of these subjects, 88 of these 288 acute diagnosed participants received cART within one month after diagnosis. The time between HIV infection and cART initiation is known to affect the size and long-term dynamics of the reservoir (43, 44), yet its effect on the factors driving persistent inflammation is less well known.

Participants were enrolled between October 2019 – October 2021. On 27 February 2020, the first COVID-19 patient was diagnosed in the Netherlands. COVID-19 epidemic was not foreseen when designing the 2000HIV study and COVID-19 infections or interventions, such as vaccinations or lock-down measurements, may affect outcome measures. All related events are therefore meticulously documented, also during the two year follow-up, and these factors will be corrected for in future analyses as they may significantly influence results. A total of 381 (20.1%) participants were enrolled before the onset of the COVID-19 pandemic. Inclusions were temporarily halted during the first COVID-19 lockdown (March-June 2020), due to local hygienic restrictions, while 1514 participants were enrolled without interruption thereafter. COVID-19 vaccination started in the Netherlands in January 2021 and was given prior to inclusion to 474 subjects, of whom 259 were fully vaccinated. Two participants did not have seroconversion after at least 14 days since being fully vaccinated. Interestingly, both were vaccinated with a viral vector vaccine (one with Janssen and one with AstraZeneca). Janssen and AstraZeneca vaccines are known to induce lower antibody titers than mRNA vaccines (45). Evidence of a previous COVID-19 infection was present at enrollment in 234 participants that were included during the COVID-19 pandemic (15.5%), three participants had been hospitalized, one of them to the ICU. Documentation of COVID-19 infection in vaccinated subjects is complicated as positive S-serology does not discern past infection from vaccination while N-serology appeared inaccurate, as described elsewhere (38).

All participants are followed up for a minimum of two years. During this period all clinical events are documented, including COVID-19. Progression of liver steatosis and fibrosis will be analyzed through repeated liver imaging. Regarding the baseline prevalence rates of psychiatric symptoms, the rates of severe symptoms of anxiety and depression (17.5%) and impulsivity (8.3%) are substantial. Most noteworthy however, are the prevalence rates of substance use during the past month: XTC was used by 8.9% during the past month, as compared to 0.8% in the general Dutch population (46). It is well known that the risk of substance use disorders, mood disorders and other mental illnesses is increased in PLHIV compared to the general population (47). In addition, nearly 17% of study participants have a high risk to develop cardiovascular diseases according to the Framingham score. Comparative cohorts have shown that cardiovascular risk is significantly increased in PLHIV over the general population, with an extensive meta-analysis showing a risk ratio for cardiovascular disease of 2.16 (95% CI, 1.68–2.77) (48). These increased risks highlight the importance of this study. By evaluating and integrating the different *omics*, we hope to unravel new pathways and biomarkers underlying the development of non-AIDS comorbidities and factors associated with the size of the HIV reservoir.

Participants were recruited from four HIV treatment centers, two of them being academic centers, in different regions throughout the Netherlands. This required overnight shipping of collected blood samples for standardized processing the next morning in a specialized lab. The well-developed infrastructure of the Netherlands and a reliable courier service facilitated efficient sample transportation. This proved to be crucial to the complex logistics of high-throughput PBMC isolation, multi-omics work-up, extensive flow cytometry and fresh ex-vivo immune stimulation experiments. To strengthen the validity of our findings in the discovery cohort (N=1559), all participants from one site form the independent validation cohort (N=336). Most baseline characteristics are similar between both sub-cohorts, except that participants in the discovery cohort have longer HIV duration (13.0 vs 10.2 years) and have been on cART longer (10.5 vs 7.7 years) than participants in the validation cohort. In this way, results can be validated in an independent cohort.

Despite a median of 10 years on cART at inclusion, and a median CD4 count of 700 cells/mm^3^, some hemocytometric parameters were different between PLHIV and healthy subjects. Although immune cell counts were not different, we found increased neutrophil and monocyte reactivity. What possibly contributes to this increased reactivity is microbial translocation, which has been attributed to play a central role in HIV immune activation. Although white blood cells in PLHIV were highly variable, HIV-specific characteristics were only modestly associated with activation parameters. Since the 2000HIV multi-omics study follows a systems biology approach, our study will help explain the variability observed within PLHIV.

## 5 Conclusion

The 2000HIV collaboration is a cohort of 1895 PLHIV that employs multi-omics to discover new biological pathways and biomarkers to unravel non-AIDS comorbidities, exceptional clinical phenotypes and factors associated with the HIV-reservoir, aiming to facilitate a future personalized approach to the best standard of care and potential cure strategies for PLHIV.

## Data availability statement

The original contributions presented in the study are included in the article/**Supplementary Materials**. Further inquiries can be directed to the corresponding author.

## Ethics statement

The studies involving human participants were reviewed and approved by Independent Review Board Nijmegen. The patients/participants provided their written informed consent to participate in this study.

## Author contributions

WV and AG wrote the manuscript. MJB, LE, AN, MJ-C, NV, VM, JCS, EM, JFr, GW, YZ, RH, RK, LV, SCV and TH wrote sections of the manuscript and gave critical revision. WV, AG, MJB and LE collected data at baseline. JFr, GW, JFu, CB, AA, JSc and LV designed the sub analyses. EW, SVV, MK, WB, KB, CR, AS, MAB, JES and AV supervised the work. QM, LJ, JL, MN and AJV designed the study and oversaw the project. All authors contributed to manuscript revision, read, and approved the submitted version.
