# Supplementary material for: The 2000HIV study: Design, multi-omics methods and participant characteristics
Source: Front Immunol. 2022 Dec 20;13:982746. doi: 10.3389/fimmu.2022.982746 (PMC9809279; doi:10.3389/fimmu.2022.982746)
Supplement: Supplementary file 1 [file DataSheet_1.docx]

**Supplementary material:**

Supplementary Table 1: Definitions of extreme clinical phenotypes (spontaneous controllers, immunological non-responders and rapid progressors)

| Non-viremic spontaneous (“elite”) controller | At least 12 months, in the absence of ART, HIV-1 RNA load <75 copies/mL, with stable CD4 count around 500 cells/mm^3^ (i.e. >75% of CD4 counts >500 cells/mm^3^) |
| --- | --- |
| Viremic spontaneous controller | At least 5 years, in the absence of ART, HIV-1 RNA load <10,000 copies/mL, with stable CD4 count around 500 cells/mm^3^ (i.e. >75% of CD4 counts >500 cells/mm^3^) |
| Rapid progressor | ≤1 year between last negative test or immunoblot confirmed acute HIV reaching CD4 <350 cells/mm^3^  OR  ≤2 years between last negative test or immunoblot confirmed acute HIV and reaching CD4 <200 cells/mm^3^ |
| Immunological non-responder | CD4 count increase <100 cells/mm^3^ in first year of cART  AND  total CD4 count <500 cells/mm^3^ after at least two years of cART |

Abbreviations: ART = Antiretroviral Therapy; mm^3^ = Cubic Millimeter; mL = milliliter; cART = Combination Antiretroviral Therapy.

Supplementary Table 2: Stimuli and measured cytokines for ex-vivo stimulation experiments using PBMCs
Supplementary Table 2A: Bacterial, viral and agonist stimuli in ex-vivo experiments using PBMCs stimulated for 24 hours.

| **Stimulus** | **Final Concentration** | **Manufacturer** | **Cat. no** |
| --- | --- | --- | --- |
| 1. Medium control | NA |  |  |
| 1. Poly I:C | 100 µg/mL | Invivogen | Tlrl-pic-5 |
| 1. LPS | 10 ng/mL | Sigma-Aldrich | L4005-100mg |
| 1. Imiquimod | 5 µg/mL | Invivogen | Tlr-imq |
| 1. Human recombinant IL-1α | 10 ng/mL | R&D Bio-techne | 200-LA-010 |
| 1. HIV-ENV | 1 µg/mL | JPT Peptide Technologies | PM-HIV-ENV |
| 1. CMV | 1 µg/mL | JPT Peptide Technologies | PM-PP65-2 |
| 1. S. pneumoniae | 5x10^6^/mL | In-house production | Strain: ATCC49619 |

Supplementary Table 2B: Measured cytokines in ex-vivo PBMC 24 hours stimulation experiment

| **Measured cytokines at 24 hours** |
| --- |
| IL-1 β |
| IL-1Ra |
| IL-6 |
| IL-8 |
| IL-10 |
| MCP-1 |
| MIP1- α |
| TNF- α |

Supplementary Table 2C: Bacterial, viral and agonist stimuli in ex-vivo experiments using PBMCs stimulated for 7 days.

| **Stimulus** | **Final Concentration** | **Manufacturer** | **Cat. no** |
| --- | --- | --- | --- |
| 1. Medium control | NA |  |  |
| 1. *E. coli* | 1x10^6^/mL | In-house production | Strain: ATCC35218 |
| 1. *S. aureus* | 1x10^6^/mL | In-house production | Strain: ATCC29213 |
| 1. *S. Pneumoniae* | 5x10^6^/mL | In-house production | Strain: ATCC49619 |
| 1. *M. tuberculosis* | 5 µg/mL | BEI Resources - H37Rv | NR-14822 |
| 1. *C. albicans* (conidia) | 1x10^6^/mL | In-house production | Strain: UC820 |
| 1. PHA | 10 µg/mL | Sigma-Aldrich | L9017-5MG |
| 1. *C. albicans* (hyphae) | 1x10^6^/mL | In-house production | Strain: UC820 |

Supplementary Table 2D: Measured cytokines in ex-vivo PBMC 7 days stimulation experiment

| Measured cytokines at 7 days |
| --- |
| IL-5 |
| IL-10 |
| IL-17 |
| IL-22 |
| IFN- γ |

Supplementary Table 3: The effect of HIV-specific factors on neutrophils, monocytes and lymphocytes is limited.

|  | **Neutrophils** | | | | | | **Monocytes** | | | | | | **Lymphocytes** | | | | | |
| --- | --- | --- | --- | --- | --- | --- | --- | --- | --- | --- | --- | --- | --- | --- | --- | --- | --- | --- |
|  | **NEUT-GI** | | **NEUT-RI** | | **NEUT-Z** | | **MO-X** | | **MO-Y** | | **MO-Z** | | **LY-X** | | **LY-Y** | | **LY-Z** | |
|  | **P value** | **β** | **P value** | **β** | **P value** | **β** | **P value** | **β** | **P value** | **β** | **P value** | **β** | **P value** | **β** | **P value** | **β** | **P value** | **β** |
| **Age** | ***0.001*** | 0.111 | 0.525 | 0.021 | 0.512 | 0.021 | ***0.006*** | 0.086 | 0.485 | 0.023 | 0.22 | 0.039 | ***<0.001*** | 0.252 | 0.145 | 0.044 | ***0.03*** | 0.068 |
| **Sex at birth** |  | | | | | | | | | | | | | | | | | |
| Male | *Reference* | - | - | - | - | - | - | - | - | - | - | - | - | - | - | - | - | - |
| Female | ***0.02*** | 0.069 | ***0.019*** | 0.071 | ***0.005*** | 0.084 | ***<0.001*** | 0.195 | 0.157 | 0.042 | ***<0.001*** | 0.147 | ***0.002*** | -0.088 | ***<0.001*** | 0.107 | ***<0.001*** | 0.203 |
| **BMI** | 0.494 | -0.019 | 0.447 | -0.022 | ***0.033*** | -0.061 | 0.845 | 0.005 | 0.063 | -0.053 | 0.08 | -0.049 | ***0.031*** | 0.058 | 0.954 | -0.002 | 0.935 | 0.002 |
| **Ethnicity** |  | | | | | | | | | | | | | | | | | |
| White | *Reference* | - | - | - | - | - | - | - | - | - | - | - | - | - | - | - | - | - |
| Asian | 0.753 | -0.009 | 0.777 | 0.008 | 0.885 | 0.004 | 0.429 | -0.021 | 0.083 | -0.048 | 0.135 | -0.041 | ***0.025*** | 0.059 | ***<0.001*** | -0.126 | 0.438 | -0.021 |
| Black | ***<0.001*** | -0.149 | 0.707 | -0.012 | 0.05 | 0.060 | ***0.001*** | 0.095 | ***0.004*** | -0.088 | ***0.03*** | -0.065 | ***<0.001*** | 0.144 | 0.977 | -0.001 | ***0.001*** | 0.095 |
| Hispanic | 0.061 | -0.052 | 0.782 | -0.008 | 0.819 | 0.006 | 0.324 | -0.027 | 0.257 | -0.032 | 0.623 | -0.013 | 0.618 | 0.013 | ***0.031*** | -0.056 | 0.133 | -0.040 |
| **Smoking** |  | | | | | | | | | | | | | | | | | |
| Never smoked | *Reference* | - | - | - | - | - | - | - | - | - | - | - | - | - | - | - | - | - |
| Current smoker | 0.569 | 0.018 | 0.391 | 0.028 | 0.343 | -0.031 | 0.71 | -0.012 | ***0.023*** | 0.074 | 0.714 | 0.012 | ***0.003*** | 0.094 | ***<0.001*** | 0.269 | ***<0.001*** | 0.119 |
| Previous smoker | 0.518 | 0.020 | 0.068 | 0.059 | 0.362 | 0.029 | 0.661 | 0.014 | 0.058 | 0.061 | 0.302 | 0.033 | ***0.003*** | 0.089 | ***<0.001*** | 0.120 | ***0.001*** | 0.101 |
| **HIV duration** | 0.512 | -0.028 | 0.207 | -0.055 | 0.119 | -0.068 | 0.195 | -0.055 | 0.49 | -0.030 | ***0.035*** | -0.091 | 0.18 | -0.055 | 0.087 | 0.070 | ***0.01*** | -0.109 |
| **cART duration** |  | | | | | | | | | | | | | | | | | |
| 0-5 years | *Reference* | - | - | - | - | - | - | - | - | - | - | - | - | - | - | - | - | - |
| 5-10 years | 0.229 | -0.055 | 0.701 | -0.018 | 0.37 | -0.041 | 0.852 | -0.008 | 0.852 | -0.008 | 0.139 | 0.068 | 0.964 | 0.002 | 0.277 | 0.047 | 0.988 | -0.001 |
| >10 years | 0.787 | -0.017 | 0.892 | -0.008 | 0.61 | -0.032 | 0.861 | 0.011 | 0.861 | 0.011 | 0.36 | 0.056 | 0.171 | 0.081 | 0.063 | 0.108 | 0.916 | -0.006 |
| **CD4 nadir** |  | | | | | | | | | | | | | | | | | |
| >500 | *Reference* | - | - | - | - | - | - | - | - | - | - | - | - | - | - | - | - | - |
| 300-500 | 0.371 | -0.042 | 0.626 | 0.023 | 0.873 | -0.007 | 0.1 | -0.076 | 0.1 | -0.076 | 0.674 | 0.019 | 0.414 | -0.036 | 0.645 | -0.020 | 0.417 | -0.037 |
| <300 | 0.526 | -0.038 | 0.884 | 0.009 | 0.829 | 0.013 | 0.448 | -0.045 | 0.448 | -0.045 | 0.472 | 0.043 | 0.86 | -0.010 | 0.825 | -0.012 | 0.562 | 0.034 |
| **VL Zenith** | 0.382 | -0.024 | 0.437 | -0.022 | 0.109 | -0.045 | 0.277 | 0.030 | 0.277 | 0.030 | ***0.012*** | -0.069 | 0.98 | 0.001 | ***0.045*** | -0.053 | 0.317 | -0.027 |
| **Latest CD4 count** | 0.555 | -0.028 | 0.612 | 0.024 | 0.054 | 0.092 | 0.179 | -0.063 | 0.179 | -0.063 | 0.062 | 0.088 | 0.91 | -0.005 | 0.097 | 0.074 | 0.637 | 0.022 |
| **Latest CD4/CD8 ratio** | 0.293 | -0.056 | 0.725 | -0.019 | 0.093 | -0.089 | 0.716 | 0.019 | 0.716 | 0.019 | 0.13 | -0.080 | ***0.005*** | -0.142 | 0.814 | -0.012 | 0.215 | -0.064 |
| **Elite controller type** |  | | | | | | | | | | | | | | | | | |
| Non-controller | *Reference* | - | - | - | - | - | - | - | - | - | - | - | - | - | - | - | - | - |
| Viremic | 0.713 | 0.011 | 0.299 | 0.031 | ***0.04*** | 0.060 | 0.585 | 0.016 | 0.585 | 0.016 | 0.254 | 0.033 | 0.15 | 0.040 | 0.096 | 0.046 | ***0.027*** | 0.063 |
| Non-viremic | 0.45 | -0.021 | ***0.025*** | 0.064 | 0.964 | -0.001 | 0.34 | 0.026 | 0.34 | 0.026 | 0.71 | -0.010 | 0.061 | 0.050 | 0.222 | 0.032 | 0.064 | 0.051 |
| **Immunological non-responders** |  | | | | | | | | | | | | | | | | | |
| No | *Reference* | - | - | - | - | - | - | - | - | - | - | - | - | - | - | - | - | - |
| Yes | 0.896 | 0.004 | 0.653 | 0.013 | 0.386 | -0.025 | 0.986 | 0.000 | 0.986 | 0.000 | 0.91 | 0.003 | 0.609 | 0.014 | 0.505 | -0.018 | 0.565 | -0.016 |
| **Rapid progressor** |  | | | | | | | | | | | | | | | | | |
| No | *Reference* | - | - | - | - | - | - | - | - | - | - | - | - | - | - | - | - | - |
| Yes | 0.248 | -0.034 | 0.991 | 0.000 | 0.175 | 0.040 | 0.108 | -0.047 | 0.108 | -0.047 | 0.188 | 0.039 | 0.073 | 0.051 | 0.581 | 0.015 | 0.224 | 0.035 |
| **Early cART** |  | | | | | | | | | | | | | | | | | |
| No | *Reference* | - | - | - | - | - | - | - | - | - | - | - | - | - | - | - | - | - |
| Yes | 0.507 | -0.019 | 0.545 | 0.018 | ***0.009*** | 0.077 | 0.086 | -0.049 | 0.889 | 0.004 | ***<0.001*** | 0.102 | ***0.029*** | -0.061 | 0.804 | -0.007 | 0.672 | 0.012 |

Univariate correlation between different hematology Sysmex variables and (non-) HIV baseline characteristics of participants in the 2000HIV study (N=1895). Definitions of elite controllers, immunological non-responders and rapid progressors can be found in Supplementary Table 1. Early cART was considered start of cART <1 months after documented acute HIV infection (incomplete immunoblot or negative hiv test within 6 months prior to diagnosis). Despite some significant p-values effect size remained limited for all parameters.

Supplementary Text 1 Hepatic steatosis and fibrosis imaging (B-mode ultrasound and FibroScan®)

*Fibroscan*®*:* The controlled attenuation parameter (CAP) and liver stiffness measurement (LSM) were measured in four hour fasting participants by universally trained operators using transient elastography (FibroScan® (Echosens, Paris, France). Participants were placed in supine position with the right arm in abduction. Measurements were made in an intercostal space at the intersection of the right midaxillary line and a transverse line at the level of the xiphoid process. In two centers the standard M probe was exclusively used, whereas in two other centers an additional XL probe was available or participants with higher BMI. Use was in accordance with instructions by the manufacturer. The measurements were considered reliable if at least 10 valid measurements were obtained, with an interquartile range (IQR) of < 30% from the median measurement. The results were expressed as kPa for liver stiffness and dB/m for CAP.

*Hepatic B-mode ultrasound:* Ultrasound examinations of the liver right lobe were performed by a trained sonographer to determine the liver stiffness (Liver Stiffness Measurement (LSM), [kPa], n=10) using shear-ware-elastography, as well as the acquisition of 2D US images (n=5) for offline determination of the steatosis driven residual attenuation coefficient (RAC, [dB/cm/MHz]), using a Mindray DC80A ultrasound system (Mindray, Shenzhen, China) with a convex probe (SC6-1E) using fixed and calibrated (1) imaging presets.

The fasting participant was placed in supine position when the probe was placed at the level of the right liver lobe. The operator scanned for images of a homogeneous part of the liver minimizing large blood vessels and bile ducts in the central part of the imaging sector for both the 2D images and liver stiffness measurements (LSM).

First, the 2D images were taken for determinations of the RAC. Around ten independent images were obtained using the following settings: [Frequency setting: 1.9~4.6 MHz; imaging depth: 13 cm; in-plane focus depth: 60mm; contrast: 36; dynamic range: 120; time-gain-compensation sliders: central position and disabling all speckle reduction options: persistence and compounding]. The five best 2D US images (most bright, least blood vessels visible) where semi-automatically processed offline to obtain the RAC using the computer aided ultrasound (CAUS) method (2-4). In short, CAUS is a generic and quantitative method that estimates ultrasound parameters, relatively to a reference phantom (ATS laboratories, tissue mimicking phantom, model 539, CIRS), enabling the estimation of the steatosis driven RAC, on 2D US images.

Second, LSM were obtained using the following settings: [Frequency setting: 1.9~4.6 MHz; imaging depth: 9 cm; in-plane focus depth: 30mm; contrast: 36 dB; dynamic range: 120; time-gain-compensation sliders: central position (using a 3D printed fixator)]. The region of interest was placed 1 cm beneath the liver capsule with a maximum of 5 cm depth. Again, large blood vessels were minimized in the region of interest. Participants were instructed to hold their breath after exhaling when the images were taken. LSM results were considered reliable if the M-STB index (indicating mobility) showed at least four stars, and the region of interest box (indicating the quality of the position) was predominantly green in color. After acquiring the image, the region of interest (diameter 15mm) was adjusted to obtain the measurement from a homogeneous area (minimal circular ROI diameter: 8mm).

After exporting the images, LSM measurements (Mean, Max, Min, SD, RLB Index, Depth, Diam) were fully automatically read from all images using optical character recognition (OCR) function (Matlab R2021a, computer vision toolbox), including automatic transfer of all values into a Microsoft Access database using ODBC connection accompanied with study information (Study-ID; Date-Time; Machine name & ID; Transducer-name; preset-name) out the DICOM header. All RAC values were also transferred automatically to the same database. Inside the database cross tables were constructed to estimate the median and IQR (p25 - p75) values from all parameters per examination.

*References Supplementary Text 1*

1. Thijssen JM, Weijers G, de Korte CL. Objective performance testing and quality assurance of medical ultrasound equipment. Ultrasound Med Biol. 2007;33(3):460-71.

2. Thijssen JM, Starke A, Weijers G, Haudum A, Herzog K, Wohlsein P, et al. Computer-aided B-mode ultrasound diagnosis of hepatic steatosis: a feasibility study. IEEE TransUltrasonFerroelectrFreqControl. 2008;55(6):1343-54.

3. Weijers G, Wanten G, Thijssen JM, van der Graaf M, de Korte CL. Quantitative Ultrasound for Staging of Hepatic Steatosis in Patients on Home Parenteral Nutrition Validated with Magnetic Resonance Spectroscopy: A Feasibility Study. Ultrasound Med Biol. 2016;42(3):637-44.

4. Munsterman ID, Groefsema MM, Weijers G, Klein WM, Swinkels DW, Drenth JPH, et al. Biochemical Effects on the Liver of 1 Month of Alcohol Abstinence in Moderate Alcohol Consumers. Alcohol and Alcoholism. 2018:agy031-agy.

Supplementary Text 2: Carotid intima-medial thickness (IMT) measurement and plaque detection:

Participants underwent high-resolution B-mode carotid artery ultrasound (Mindray DC80a ultrasound, Shenzhen, China) with a 4.0-12.6 MHz transducer. Intima-media thickness (IMT) was measured in the far wall, 1 cm distal of the carotid bulb in the left and right common carotid artery (CCA). All researchers were uniformly trained in the Radboud University Medical Centre. A plaque was defined as a focal IMT > 1.5mm or thickening of the IMT > 50% compared to the mean IMT in the common carotid artery, carotid bulb or in the internal carotid artery (ICA).

Supplementary Text 3: Electrocardiography

A 12-lead resting ECG was recorded by MAC600 electrocardiography (GE Healthcare, Chicago, United States) at a sample frequency of 500Hz and digitally stored. ECGs were processed by Modular ECG analysis system (MEANS) to obtain standardized interpretations according to the Minnesota Code (1,2).

*References Supplementary Text 3*

1. Prineas RJCRSBHW. The Minnesota code manual of electrocardiographic findings: standards and procedures for measurement and classification. Boston, Mass.: J. Wright; 1982.
2. van Bemmel JH, Kors JA, van Herpen G. Methodology of the modular ECG analysis system MEANS. Methods Inf Med. 1990;29(4):346-53.

Supplementary text 4: Extensive -omics methods description

In the 2000HIV study, the following layers of *-omics* data are analyzed: genomics, epigenomics, transcriptomics, metabolomics, proteomics, and stool and saliva metagenomics. For each layer of *-omics* data, strict quality control filters are applied (e.g., removing outlier samples), followed by proper pre-processing and normalization. Multi-omics analyses are still ongoing at the time of writing.

*4.1 Multi-omics methods*

*4.1.1 Genomics*

To identify single nucleotide polymorphisms (SNPs), DNA samples obtained from whole blood of the participants are genotyped using the commercially available genome-wide SNP array (Illumina Infinium Global Screening Array). Genotype data are imputed using the human reference consortium (HRC) as a reference panel (1), and appropriate quality control filters per sample and SNP are applied before follow-up analysis. Next, to investigate whether common genetic polymorphisms (minor allele frequency ≥5%) influence the immune traits (cytokine production capacity in response to various stimuli, immune cell subpopulations and immunoglobulin subclasses) the correlation between SNPs and the variation of these traits are tested using quantitative trait locus (QTL) mapping as previously described (2). In addition, a whole genome sequencing approach is used in a subset of 200 individuals with a range of rare clinical cases and extreme clinical phenotypes to identify a genetic cause, especially rare genetic variants, underlying these phenotypes.

*4.1.2 Epigenomics*

Genome-wide profiling of chromatin accessibility landscapes is performed using the Assay for Transposase-Accessible Chromatin using sequencing (ATAC-seq) (3), on participant PBMCs and isolated CD4+ T-cells, CD8+ T-cells, B-cells, NK-cells and monocytes. These cell subsets are isolated using positive magnetic bead selection targeted at CD4, CD8, CD56, CD19 and CD14 respectively. For each sample, ~50,000 cells are tagmented. To construct the libraries for Illumina single-end sequencing, samples are pooled and amplified according to the obtained amount of DNA as estimated by a quantitative PCR (qPCR) reaction. Data processing and unsupervised analysis steps are followed using a publicly available pipeline on Zenodo and GitHub (4). In brief, several quality control metrics are aggregated and reported using MultiQC (5). Next, regions of the genome are scanned for reads enrichments, creating a consensus region set. For each of these regions, the ATAC-seq signal intensity is quantified in each sample. Unsupervised analyses are performed using principal component analysis and Uniform Manifold Approximation and Projection (UMAP) (6) to reduce the data dimensionality and provide two-dimensional visualization. Finally, supervised analyses are performed using limma-voom (7, 8) and DESeq2 (9).

*4.1.3 Transcriptomics*

For transcriptomics analysis, bulk RNA sequencing of PBMCs is performed by short read sequencing using current Illumina technology (>30 million reads per sample). The sequencing reads are mapped to the human reference genome NCBI build 38 or later current version using STAR alignment. Gene expression is estimated using HTSeqCount function from DESeq2 using the most recent version of Ensembl gene annotation. Raw counts are passed to the DESeq2 pipeline (9) which includes rlog transformation, normalization and exclusion of low abundant transcripts. Next, to identify differentially expressed genes, differential expression analysis is performed by stratifying the participants into different groups by clinical phenotype. To identify the structure in the cohort based on transcriptome data, co-expression networks are constructed using CoCena² (10). Patterns in the resulting gene expression modules stratify the individuals according to their molecular phenotype. These data-driven subgroups are then investigated in relation to other -*omics* and clinical data layers. Further analyses include pathway enrichment analysis to identify molecular pathways and transcription factor motif enrichment analysis help identifying important regulatory hubs. In addition to bulk RNA sequencing, single-cell RNA sequencing is performed for a subset of 200 individuals with rare clinical phenotypes using BD Rhapsody^TM^ Single-cell Analysis System (BD Biosciences, Franklin Lakes, New Jersey US) to create single-cell suspensions for RNA sequencing as described elsewhere (11, 12).

*4.1.4 Metabolomics*

Untargeted metabolome profiling is performed on plasma samples using flow injection electrospray – time-of-flight mass spectrometry, according to the methodology described previously (13). The duplicate peak intensity of raw metabolome data is averaged and normalized prior to analysis. Principal component analysis is then applied to identify possible outlier samples. Metabolites are annotated and further categorized based on metabolomic source (endogenous, food, or drugs) and chemical taxonomy using publicly available data from The Human Metabolome Database (14). To identify metabolites and metabolic pathways associated with specific phenotypes, linear regression analysis will be performed, followed by over-representation and pathway enrichment analysis in the MetaboAnalyst web-platform (http://www.metaboanalyst.ca/) (15).

*4.1.5 Proteomics*

Circulating plasma protein expression is assessed with the use of a commercially available multiplex proximity extension assay (PEA) from Olink® proteomics AB (Uppsala, Sweden), as described elsewhere (16). In brief, in the PEA proteins are recognized by pairs of oligonucleotide-labeled antibodies (“PEA probes”), which are used to bind target proteins in a pairwise manner. Upon binding, the oligonucleotides come in close proximity and hybridize followed by extension using a polymerase chain reaction. The sequence is quantified by quantitative real-time PCR and the number of PCR templates is proportional to the protein concentration in the plasma. PEA allows an increased number of 384 multiplex assays and higher throughput using next-generation sequencing (NGS) as a readout method (16). For this study, we use the full library (Olink® Explore 3072) consisting of 3072 targeted proteins divided into eight 384-plex panels focused on inflammatory, oncological, cardiometabolic and neurological proteins. Protein measurements are delivered as Normalized Protein Expression (NPX) values through a quality control and normalization process developed and provided by Olink (16). NPX values are Cq values normalized by the subtraction of values for extension control as well as the plate control (plasma sample). The scale is shifted using a correction factor (normal background noise) and reported in the Log_2_ scale. To assess the correlation structure of proteins and identify possible endophenotypes within PLHIV, unsupervised hierarchical clustering is performed. Further, to test which proteins are differentially expressed between PLHIV with different phenotypes, a differential expression analysis is performed using a linear regression model with appropriate variables as covariates such as age and/or sex at birth. The identified differentially expressed proteins are subjected to further downstream analysis for obtaining more biological insight using pathway enrichment analysis on Metascape web-platform (http://metascape.org/) (17). For biomarker identification on specific phenotypes, machine learning approaches are followed using commonly used penalized regression methods, such as ridge, lasso or elastic net regression. The classification model’s performance are evaluated using the receiver operating characteristic (ROC) curve.

*4.1.6 Metagenomics*

Fecal and saliva DNA is whole genome shotgun sequenced using an input DNA of 200 – 220 ng from stool and saliva samples for library preparation using the MGIEasy FS DNA Library Prep Set (item number 1000017572). Metagenomic sequencing is performed on the DNBSEQ-T7 platform, with 150 bp of paired-end reads for all samples. For stool samples, 20 million reads, 6 Gb raw bases per sample is generated, whilst for saliva samples, 50 million reads, 15 Gb raw bases per sample is generated. Microbial taxonomic profiles is determined using MetaPhlAn3 (v3.0.7) (18). Functional profiling is performed using HUMAnN3 (v3.0.0.alpha.3) (18) by mapping the taxonomic profiling to species-specific pangenomes, with UniRef90 annotations. Finally, the strain level profiling of gene composition, SNPs, structural variations are assessed using Pangenome-based Phylogenomic Analysis3 (PanPhlAn3) (18), GenoTyper for Prokaryotes (GT-Pro) (19), and SVFinder (20), respectively.

*4.2 Immune cells and their function*

*4.2.1 Flowcytometric immunophenotyping (CytoFLEX)*

Whole blood samples are immunophenotyped by using three flow cytometry panels containing 17-20 markers each and custom-made tubes with dry antibodies from DURA Innovations Technology (Beckman Coulter). The panels are aimed to identify the cell proportions of the innate and the adaptive immune system compartments, as well as the expression of key markers related with activation, exhaustion, and the evaluation of T- and B-cells developmental stages (21). Cells are acquired in a twenty one-color, six- laser CytoFLEX-LX (Beckman Coulter) and using Cytexpert software 2.3 (Beckman Coulter). Instrument quality control and standardization are performed daily using CytoFLEX Daily QC Fluorospheres (Beckman Coulter catalog #B53230), CytoFLEX Daily IR QC Fluorospheres beads (Beckman Coulter catalog # C06147) and SPHEROtm Rainbow calibration particles 6-peak (Spherotech Inc, catalog # RCP-30-5A-6) (21). Data analysis is performed using Kaluza V2.1.2 and Cytobank Platform V9.0 (Beckman Coulter).

*4.2.2 Ex-vivo cytokine production capacity*

To measure cytokine production capacity, PBMCs are stimulated with a variety of whole (inactivated) pathogens, pattern recognition receptor ligands, other pathogen-derived antigens, and viral stimuli (Supplementary Table 2A and 2C). The stimulations are performed with 500,000 cells per well in round-bottom 96-wells plates (Greiner) for either 7 days (in the presence of 10% human pool serum) or 24 hours at 37⁰C and 5% CO_2_. Supernatants are collected and stored at -20⁰C until measurement of relevant cytokines with ELISA (Supplementary Table 2B and 2D).

*4.3 HIV latent reservoir size and composition*

HIV-1 DNA (total and intact) and HIV-1 RNA (total and long LTR) are measured by digital PCR (dPCR) in isolated CD4^+^ T-cells (22-25). Starting from 40 million cryopreserved PBMCs, CD4^+^ T-cells are automatically isolated by negative selection, using the EasySep Human CD4^+^+ T-cell isolation kit on the Robosep-S (Stemcell Technologies, Vancouver, Canada). CD4^+^ T-cells are aliquoted in two; genomic DNA is extracted using the QiaAmp DNA mini kit on the Qiacube (Qiagen, Hilden, Germany); RNA is extracted using the RNeasy mini kit, including DNase step on the Qiacube (Qiagen, Hilden, Germany). Nucleic acid concentrations is determined with the Qubit™ DNA BR Assay Kit or Qubit™ RNA BR Assay Kit, by using the Qubit fluorometer (Thermofisher Scientific, Waltham, Massachusetts, United States). cDNA synthesis is performed immediately following RNA extraction (SuperScript III; Invitrogen by Thermofisher Scientific, Waltham, Massachusetts, United States) as described by Yukl et al.(24). DNA and RNA is quantified in triplicate by dPCR using different primer/probe sets. DNA is normalized by measuring the reference gene RPP30 in duplicate by ddPCR and expressed per million CD4^+^ T-cells. For HIV-1 RNA normalization, three reference genes (B2M, GAPDH and ACTB) are measured. HIV-1 RNA copies are divided by the geometric mean of the reference genes and expressed per million CD4^+^ T-cells. Moreover, in spontaneous controllers the proviral landscape is analyzed by FLIP-Seq (Full-length Individual Proviral Sequencing) at single genome resolution (26).

*References supplementary text 4*

1. McCarthy S, Das S, Kretzschmar W, Delaneau O, Wood AR, Teumer A, et al. A Reference Panel of 64,976 Haplotypes for Genotype Imputation. *Nat Genet* (2016) 48(10):1279-83. Epub 20160822. doi: 10.1038/ng.3643.

2. Li Y, Oosting M, Smeekens SP, Jaeger M, Aguirre-Gamboa R, Le KTT, et al. A Functional Genomics Approach to Understand Variation in Cytokine Production in Humans. *Cell* (2016) 167(4):1099-110 e14. doi: 10.1016/j.cell.2016.10.017.

3. Buenrostro JD, Giresi PG, Zaba LC, Chang HY, Greenleaf WJ. Transposition of Native Chromatin for Fast and Sensitive Epigenomic Profiling of Open Chromatin, DNA-Binding Proteins and Nucleosome Position. *Nat Methods* (2013) 10(12):1213-8. Epub 20131006. doi: 10.1038/nmeth.2688.

4. S. Reichl BE, D. Barreca, L. Folkman, L. Dobnikar, C. Bock. Ultimate Atac-Seq Data Processing & Analysis Pipeline: Zenodo (2022) https://zenodo.org/record/6323635#.YrmHQnZBwYs [Accessed June 20, 2022].

5. Ewels P, Magnusson M, Lundin S, Kaller M. Multiqc: Summarize Analysis Results for Multiple Tools and Samples in a Single Report. *Bioinformatics* (2016) 32(19):3047-8. Epub 20160616. doi: 10.1093/bioinformatics/btw354.

6. McInnes L, Healy J, Saul N, Großberger L. Umap: Uniform Manifold Approximation and Projection. *Journal of Open Source Software* (2018) 3(29):861. Epub 02 September 2018. doi: 10.21105/joss.00861.

7. Smyth GK. Limma: Linear Models for Microarray Data. In: Gentleman R. CVJ, Huber W., Irizarry R.A., Dudoit S., editor. *Bioinformatics and Computational Biology Solutions Using R and Bioconductor*. Statistics for Biology and Health. Springer, New York, NY (2005). p. 397-420.

8. Law CW, Chen Y, Shi W, Smyth GK. Voom: Precision Weights Unlock Linear Model Analysis Tools for Rna-Seq Read Counts. *Genome Biol* (2014) 15(2):R29. Epub 20140203. doi: 10.1186/gb-2014-15-2-r29.

9. Love MI, Huber W, Anders S. Moderated Estimation of Fold Change and Dispersion for Rna-Seq Data with Deseq2. *Genome Biol* (2014) 15(12):550. doi: 10.1186/s13059-014-0550-8.

10. Aschenbrenner AC, Mouktaroudi M, Kramer B, Oestreich M, Antonakos N, Nuesch-Germano M, et al. Disease Severity-Specific Neutrophil Signatures in Blood Transcriptomes Stratify Covid-19 Patients. *Genome Med* (2021) 13(1):7. Epub 20210113. doi: 10.1186/s13073-020-00823-5.

11. Shum EY, Walczak EM, Chang C, Christina Fan H. Quantitation of Mrna Transcripts and Proteins Using the Bd Rhapsody Single-Cell Analysis System. *Adv Exp Med Biol* (2019) 1129:63-79. doi: 10.1007/978-981-13-6037-4_5.

12. Markello TC, Carlson-Donohoe H, Sincan M, Adams D, Bodine DM, Farrar JE, et al. Sensitive Quantification of Mosaicism Using High Density Snp Arrays and the Cumulative Distribution Function. *Mol Genet Metab* (2012) 105(4):665-71. Epub 20111224. doi: 10.1016/j.ymgme.2011.12.015.

13. Fuhrer T, Heer D, Begemann B, Zamboni N. High-Throughput, Accurate Mass Metabolome Profiling of Cellular Extracts by Flow Injection-Time-of-Flight Mass Spectrometry. *Anal Chem* (2011) 83(18):7074-80. Epub 20110818. doi: 10.1021/ac201267k.

14. Wishart DS, Feunang YD, Marcu A, Guo AC, Liang K, Vazquez-Fresno R, et al. Hmdb 4.0: The Human Metabolome Database for 2018. *Nucleic Acids Res* (2018) 46(D1):D608-D17. doi: 10.1093/nar/gkx1089.

15. Pang Z, Chong J, Zhou G, de Lima Morais DA, Chang L, Barrette M, et al. Metaboanalyst 5.0: Narrowing the Gap between Raw Spectra and Functional Insights. *Nucleic Acids Res* (2021) 49(W1):W388-W96. doi: 10.1093/nar/gkab382.

16. Filbin MR, Mehta A, Schneider AM, Kays KR, Guess JR, Gentili M, et al. Longitudinal Proteomic Analysis of Severe Covid-19 Reveals Survival-Associated Signatures, Tissue-Specific Cell Death, and Cell-Cell Interactions. *Cell Rep Med* (2021) 2(5):100287. Epub 20210503. doi: 10.1016/j.xcrm.2021.100287.

17. Zhou Y, Zhou B, Pache L, Chang M, Khodabakhshi AH, Tanaseichuk O, et al. Metascape Provides a Biologist-Oriented Resource for the Analysis of Systems-Level Datasets. *Nat Commun* (2019) 10(1):1523. Epub 20190403. doi: 10.1038/s41467-019-09234-6.

18. Beghini F, McIver LJ, Blanco-Miguez A, Dubois L, Asnicar F, Maharjan S, et al. Integrating Taxonomic, Functional, and Strain-Level Profiling of Diverse Microbial Communities with Biobakery 3. *Elife* (2021) 10. Epub 20210504. doi: 10.7554/eLife.65088.

19. Shi ZJ, Dimitrov B, Zhao C, Nayfach S, Pollard KS. Fast and Accurate Metagenotyping of the Human Gut Microbiome with Gt-Pro. *Nat Biotechnol* (2022) 40(4):507-16. Epub 20211223. doi: 10.1038/s41587-021-01102-3.

20. Zeevi D, Korem T, Godneva A, Bar N, Kurilshikov A, Lotan-Pompan M, et al. Structural Variation in the Gut Microbiome Associates with Host Health. *Nature* (2019) 568(7750):43-8. Epub 20190327. doi: 10.1038/s41586-019-1065-y.

21. Navas A, Van de Wijer L, Jacobs-Cleophas M, Schimmel-Naber AM, van Cranenbroek B, van der Heijden WA, et al. Comprehensive Phenotyping of Circulating Immune Cell Subsets in People Living with Hiv. *Journal of Immunological Methods* (2022):*in press*. doi: 10.1016/j.jim.2022.113307.

22. Rutsaert S, Bosman K, Trypsteen W, Nijhuis M, Vandekerckhove L. Digital Pcr as a Tool to Measure Hiv Persistence. *Retrovirology* (2018) 15(1):16. Epub 20180130. doi: 10.1186/s12977-018-0399-0.

23. Hindson BJ, Ness KD, Masquelier DA, Belgrader P, Heredia NJ, Makarewicz AJ, et al. High-Throughput Droplet Digital Pcr System for Absolute Quantitation of DNA Copy Number. *Anal Chem* (2011) 83(22):8604-10. Epub 20111028. doi: 10.1021/ac202028g.

24. Yukl SA, Kaiser P, Kim P, Telwatte S, Joshi SK, Vu M, et al. Hiv Latency in Isolated Patient Cd4(+) T Cells May Be Due to Blocks in Hiv Transcriptional Elongation, Completion, and Splicing. *Sci Transl Med* (2018) 10(430). doi: 10.1126/scitranslmed.aap9927.

25. Bruner KM, Wang Z, Simonetti FR, Bender AM, Kwon KJ, Sengupta S, et al. A Quantitative Approach for Measuring the Reservoir of Latent Hiv-1 Proviruses. *Nature* (2019) 566(7742):120-5. Epub 20190130. doi: 10.1038/s41586-019-0898-8.

26. Lee GQ, Orlova-Fink N, Einkauf K, Chowdhury FZ, Sun X, Harrington S, et al. Clonal Expansion of Genome-Intact Hiv-1 in Functionally Polarized Th1 Cd4+ T Cells. *J Clin Invest* (2017) 127(7):2689-96. Epub 20170619. doi: 10.1172/JCI93289.

Supplementary Text 5: methods of COVID-19 serology

At baseline and two-year follow-up COVID-19 serology will be measured. In addition to Spike (S)-, also Nucleocapsid (N)-serology will be measured in attempt to discern infection from vaccination. Participants enrolled in our study before 12-03-2020 (i.e. last date of inclusions before lockdown) are considered non-COVID-19 exposed subjects (negative controls). A proven COVID-19 infection was considered when participants had a documented positive PCR test or had positive S-serology measured during routine care in participating hospitals (positive controls). For S-serology only participants without a COVID-19 vaccination can be used as positive controls due to the effect of vaccination on serum S-antibody levels. For N-serology the same criteria apply regardless of COVID-19 vaccination, as current vaccines have no effect on N-serology. In brief, for both S- and N- serology, cut-off values for positive serology will be determined using receiver operating characteristic with a false positive rate of <2% and a true positive rate of >90%. Seroprevalence studies from two neighboring countries show that by end of March 2020 the seroprevalence was <2% (1,2). Data analysis will be done in R-studio version 1.4.1106, using “ggplot2” and “ROCit” packages. Log2 transformation will be used for the serum antibody levels. Antibody levels below or above the limit of detection are imputed as the limit of detection values.
Lastly, we will count the number of past COVID-19 infections in our study. A past COVID-19 infection was defined as having had either positive S-serology measured at baseline as described above, a reported positive PCR test in the past or a positive S-serology measurement in their local hospital as part of routine care.

*References Supplementary Text 5*

1. Herzog SA, De Bie J, Abrams S, Wouters I, Ekinci E, Patteet L, et al. Seroprevalence of IgG antibodies against SARS-CoV-2 - a serial prospective cross-sectional nationwide study of residual samples, Belgium, March to October 2020. Euro Surveill. 2022;27(9).
2. Le Vu S, Jones G, Anna F, Rose T, Richard JB, Bernard-Stoecklin S, et al. Prevalence of SARS-CoV-2 antibodies in France: results from nationwide serological surveillance. Nat Commun. 2021;12(1):3025.

Supplementary Text 6: Results of COVID-19 serology

At baseline, for S-serology an AUC = 0.9844 was identified (supplementary figure 1A). The threshold for positive serology was set at 14.41 IU/ml giving a true positive rate (TPR) of 93.5% and a false positive rate (FPR) of 1.8% (supplementary figure 1B). When we recalculated the ROC restricting negative controls to sampling before 27 February 2020 (first proven COVID-19 case in the Netherlands) or before 1 January 2020, this had no significant impact on the AUC (0.98 and 0.99, respectively). N-serology analysis was used with the aim to identify infections in the vaccinated group. However, antibody levels in the negative controls did not substantially differ from the positive controls (AUC = 0.8797), resulting in a too low sensitivity and specificity to enable robust distinction (supplementary figure 1A and 1C).


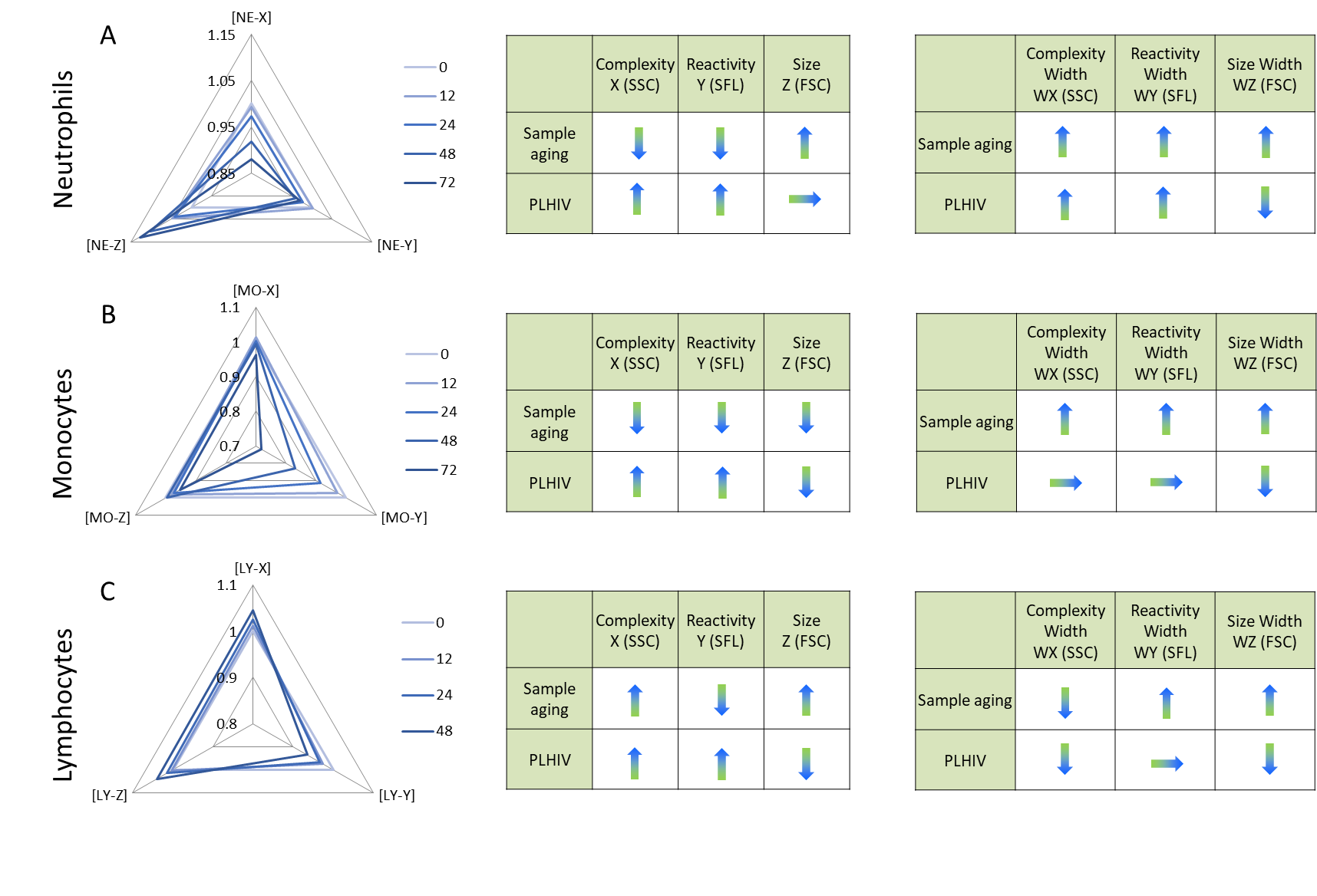
Supplementary Figure 1: PLHIV samples were processed after overnight shipping, whereas the healthy controls were processed within 6 hours. Although sample aging slightly affected the white blood cell characteristics, these changes are not consistent with the differences observed between PLHIV and healthy controls. Width represents the intra-individual variability per cell type on each axis. The triangles show changes observed in sample aging and the tables display the changes next to changes observed in PLHIV versus healthy controls, for neutrophils (A), monocytes (B), lymphocytes (C).

Supplementary Figure 2A: Presence or absence in serum of anti-CMV-antibody IgG between discovery and validation cohorts within the 2000HIV study. Anti-CMV IgG seropositivity as measured with ELISA, compared through chi-squared test. Number of PLHIV in Discovery cohort = 1559 PLHIV. Number of PLHIV on Validation cohort; N = 336.


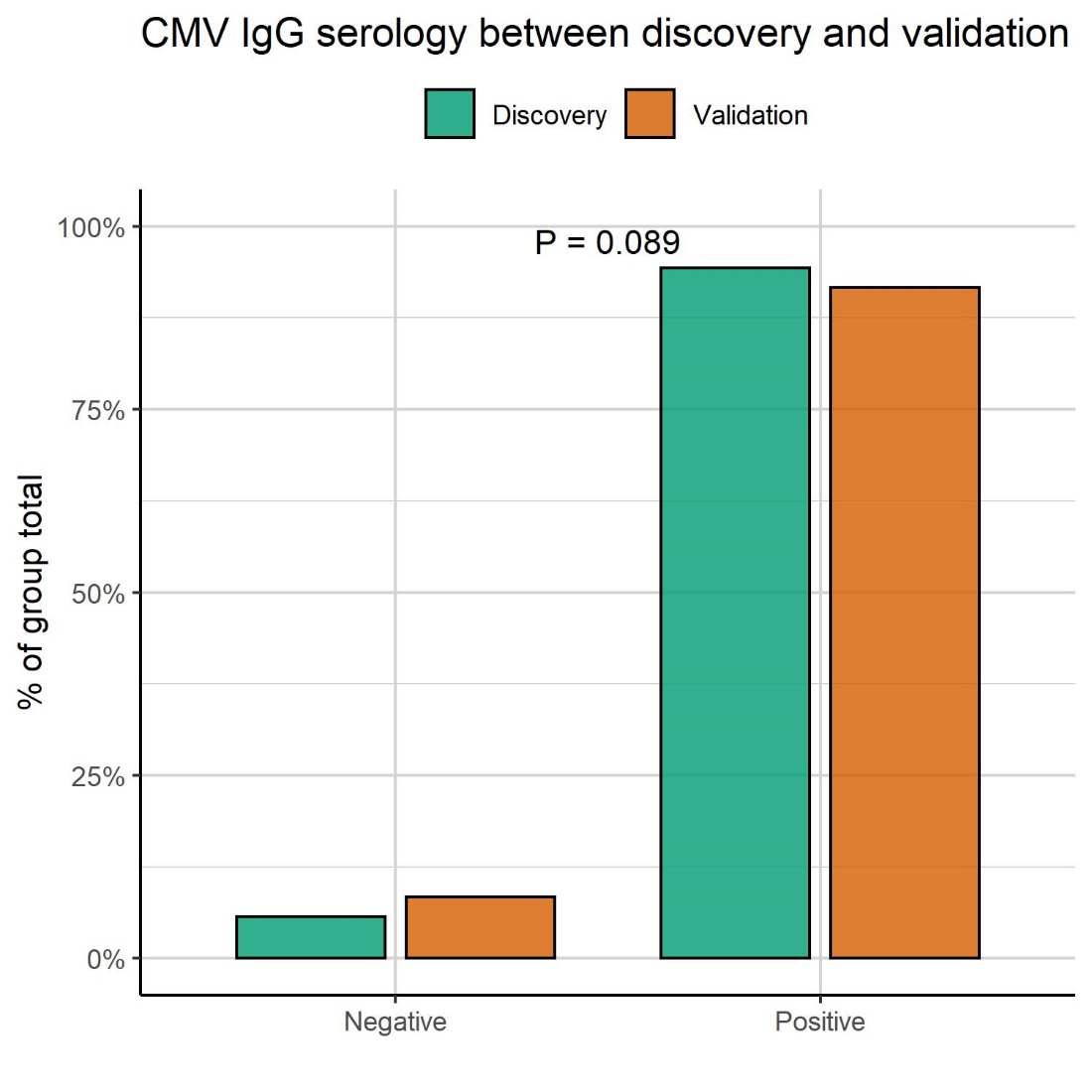


Abbreviation: CMV = Cytomegalovirus

Supplementary Figure 2B: Antibody levels of serum anti-CMV IgG between discovery and validation cohorts within the 2000HIV study Anti-CMV IgG serology titers as measured with ELISA, compared through Wilcoxon Rank Sum test. Number of PLHIV in Discovery cohort = 1559 PLHIV. Number of PLHIV on Validation cohort; N = 336.


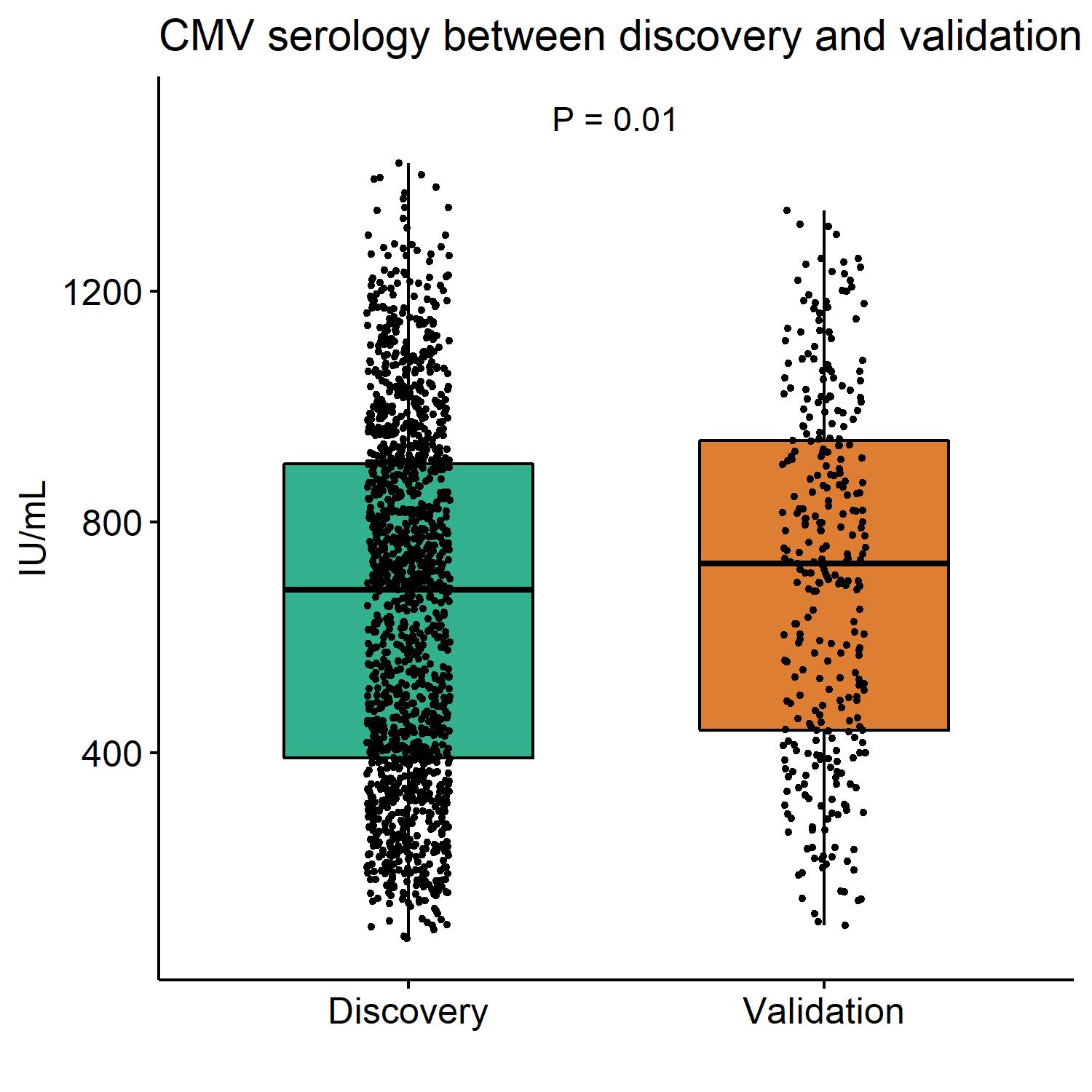


Abbreviation: CMV = Cytomegalovirus

Supplementary Figure 3: Supporting figures for COVID-19 serology.

Supplementary Figure 3A: Receiver operating characteristic SARS-CoV-2 anti-S- and anti-N-protein IgG antibodies with area under the curve values. Anti-S-protein antibody levels are able to distinguish infection in unvaccinated participants. Anti-N-protein antibody level sensitivity and specificity remain too low to distinguish infection in vaccinated people accurately. Number of PLHIV in negative controls for both anti-S and anti-N = 379, number of PLHIV in positive controls anti-S = 62, number of PLHIV in positive controls anti-N = 117.


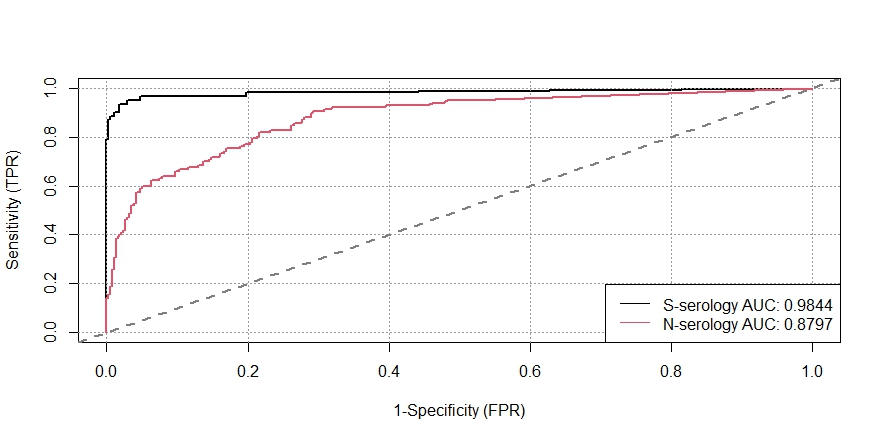


Abbreviations: S = Spike, N = Nucleocapsid, AUC = area under the curve, TPR = true positive rate, FPR = false positive rate.

Supplementary Figure 3B: SARS-CoV-2 anti-S-protein antibody levels in positive and negative controls with cut-off point for positivity as calculated through receiver operating characteristic analysis. Anti-S-protein antibody levels are in log_2_ scale. Number of PLHIV in negative controls = 379, number of PLHIV in positive controls = 62.


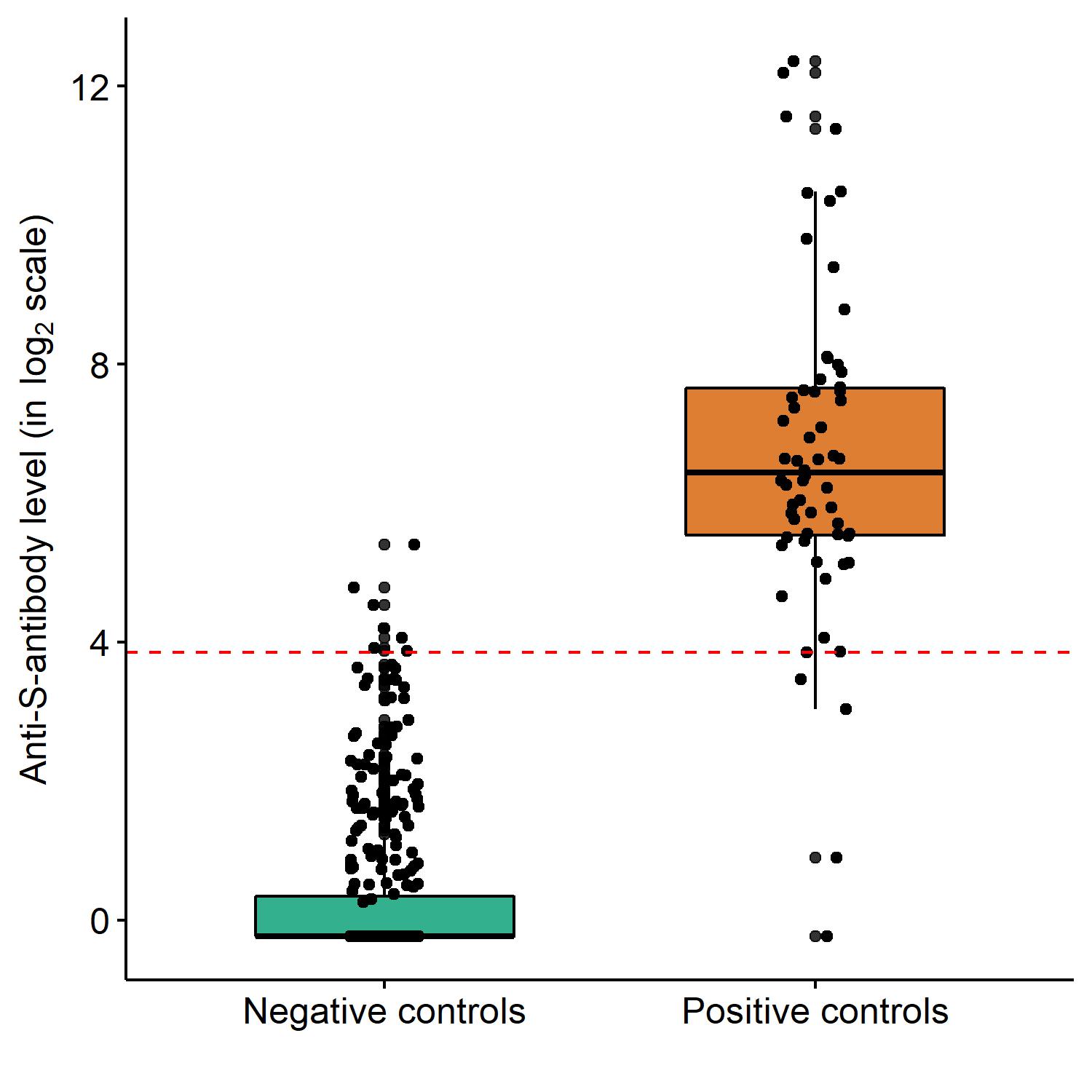


Abbreviation: S = Spike

Supplementary Figure 3C: SARS-CoV-2 anti-N-protein antibody levels in positive and negative controls. Overlap between positive and negative controls prevented a reliable cut-off value to be established through receiver operating characteristic analysis. Anti-N-protein antibody levels are in log_2_ scale. Number of PLHIV in negative controls = 379, number of PLHIV in positive controls = 117.


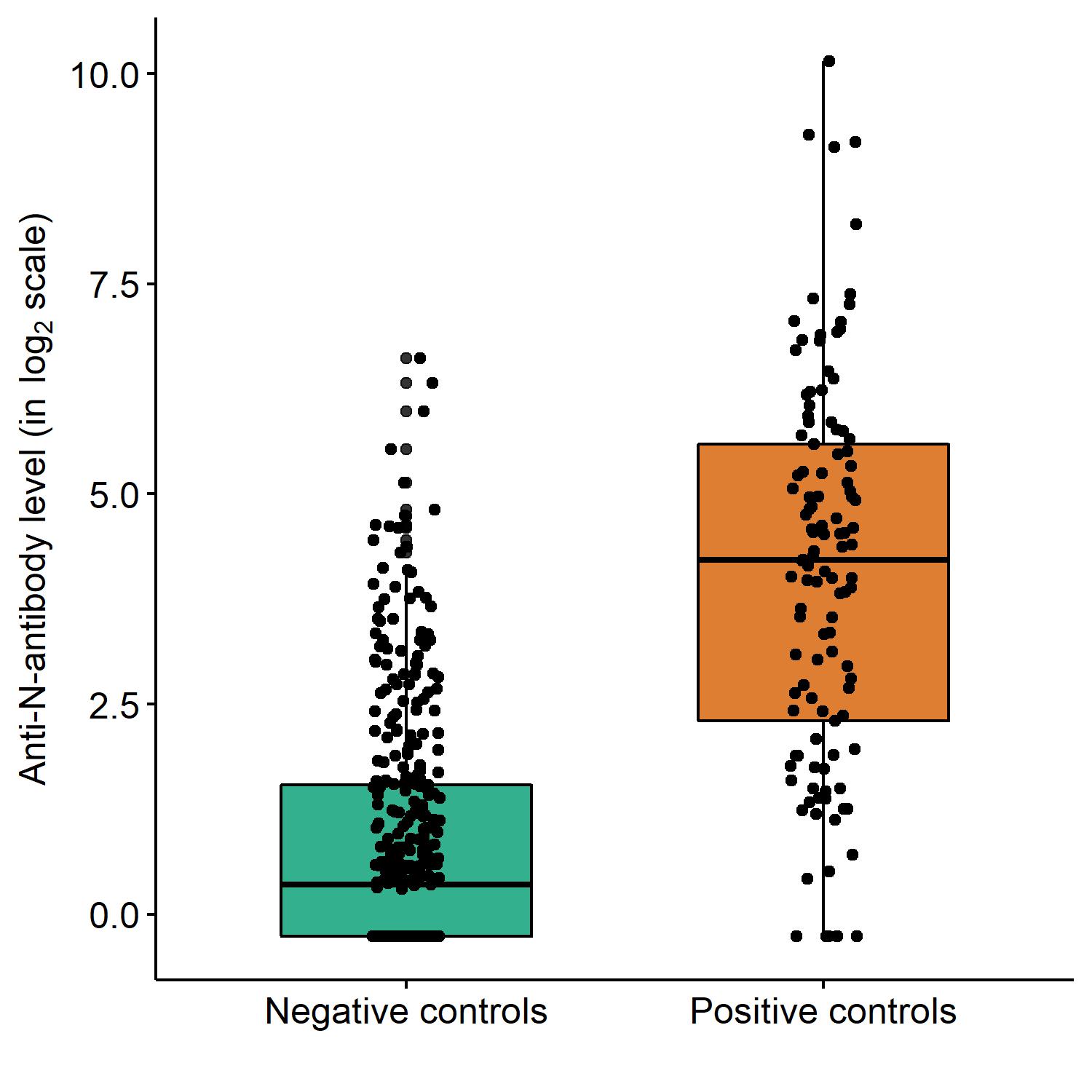


Abbreviations: N = Nucleocapsid


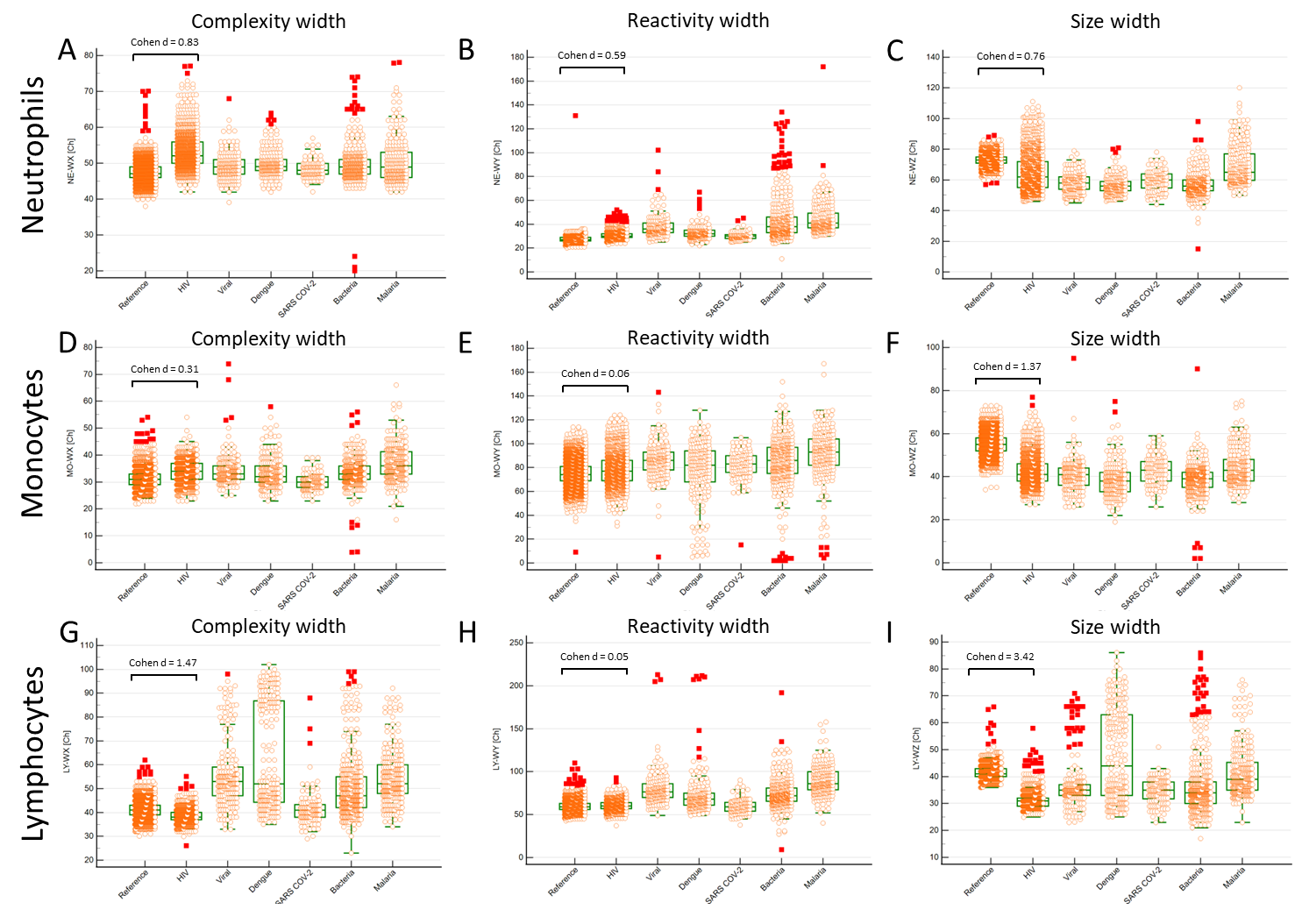
Supplementary Figure 4: Intra-individual variability (width) in neutrophil, monocyte and lymphocyte characteristics were compared between N = 1895 PLHIV with suppressed viral loads and N = 15,803 people without HIV. To place treated HIV in a context, PLHIV were displayed next to acute infectious disease cohorts. These other cohorts consisted of patients presenting with acute febrile disease at the ER who subsequently underwent extensive diagnostic testing. Cohen d displays the effect size. Differences were considered significant if P < 0.05 and relevant if Cohen d effect size was > 0.5 **A.-C.** Neutrophils of PLHIV had greater intra-individual variability in complexity and reactivity, yet smaller in size, similar to acute infectious diseases. **D.-F.** Monocytes of PLHIV were more variable in size, yet not in complexity and reactivity. **G.-I.** Lymphocytes of PLHIV displayed smaller variability in complexity and size, but not in reactivity.
