# Supplementary material for: The 2000HIV study: Design, multi-omics methods and participant characteristics
Source: Front Immunol. 2022 Dec 20;13:982746. doi: 10.3389/fimmu.2022.982746 (PMC9809279; doi:10.3389/fimmu.2022.982746)
Supplement: Supplementary file 2 [file DataSheet_2.docx]

**All contributors to the ATHENA cohort:**

**Clinical centres:**** denotes site coordinating physician*

**Amsterdam UMC, AMC site, Amsterdam:**

HIV treating physicians: M. van der Valk*, S.E. Geerlings, A. Goorhuis, V.C. Harris, J.W. Hovius, B. Lempkes, F.J.B. Nellen, T. van der Poll, J.M. Prins, V. Spoorenberg, M. van Vugt, W.J. Wiersinga, F.W.M.N. Wit. HIV nurse consultants: C. Bruins, J. van Eden, A.M.H. van Hes, F.J.J. Pijnappel, S.Y. Smalhout, A.M. Weijsenfeld. HIV clinical virologists/chemists: N.K.T. Back, B. Berkhout, M.T.E. Cornelissen, R. van Houdt, M. Jonges, S. Jurriaans, C.J. Schinkel, K.C. Wolthers, H.L. Zaaijer.

**Amsterdam UMC, VUmc site, Amsterdam:**

HIV treating physicians: E.J.G. Peters*, M.A. van Agtmael, R.S. Autar, M. Bomers, K.C.E. Sigaloff. HIV nurse consultants: M. Heitmuller, L.M. Laan. HIV clinical virologists/chemists: N.K.T. Back, B. Berkhout, M.T.E. Cornelissen, R. van Houdt,

M. Jonges, S. Jurriaans, C.J. Schinkel, K.C. Wolthers, H.L. Zaaijer.

**Emma Kinderziekenhuis (Amsterdam UMC, AMC site), Amsterdam:**

HIV treating physicians: M. van der Kuip, D. Pajkrt. HIV nurse consultants: F.M. Hessing, A.M. Weijsenfeld.

**Admiraal De Ruyter Ziekenhuis, Goes:**

HIV treating physicians: M. van den Berge*, A. Stegeman. HIV nurse consultants: S. Baas,

L. Hage de Looff. HIV clinical virologists/chemists: A. van Arkel, J. Stohr, B. Wintermans.

**Catharina Ziekenhuis, Eindhoven:**

HIV treating physicians: M.J.H. Pronk*, H.S.M. Ammerlaan. HIV nurse consultants: E.S. de Munnik. HIV clinical virologists/chemists: B. Deiman, A.R. Jansz, V. Scharnhorst, J. Tjhie, M.C.A. Wegdam.

**DC Klinieken Lairesse – Hiv Focus Centrum, Amsterdam:**

HIV treating physicians: M. van der Valk*, A. van Eeden, E. Hoornenborg, J. Nellen.

HIV nurse consultants: W. Alers, L.J.M. Elsenburg, H. Nobel. HIV clinical virologists/chemists:C.J. Schinkel.

**ETZ (Elisabeth-TweeSteden Ziekenhuis), Tilburg:**

HIV treating physicians: M.E.E. van Kasteren*, M.A.H. Berrevoets, A.E. Brouwer. HIV nurse specialist: B.A.F.M. de Kruijf-van de Wiel. HIV nurse consultants: A. Adams, M. Pawels-van Rijkevoorsel. HIV data collection: B.A.F.M. de Kruijf-van de Wiel. HIV clinical virologists/chemists: A.G.M. Buiting, J.L. Murck.

**Erasmus MC, Rotterdam:**

HIV treating physicians: C. Rokx*, A.A. Anas, H.I. Bax, E.C.M. van Gorp, M. de Mendonça Melo, E. van Nood, J.L. Nouwen, B.J.A. Rijnders, C.A.M. Schurink, L. Slobbe, T.E.M.S. de Vries-Sluijs. HIV nurse consultants: N. Bassant, J.E.A. van Beek, M. Vriesde, L.M. van Zonneveld. HIV data collection: J. de Groot. HIV clinical virologists/chemists: J.J.A. van Kampen, M.P.G Koopmans, J.C. Rahamat-Langendoen.

**Erasmus MC Sophia Kinderziekenhuis, Rotterdam:**

HIV treating physicians: P.L.A. Fraaij, A.M.C. van Rossum, C.L. Vermont. HIV nurse consultants: L.C. van der Knaap.

**Flevoziekenhuis, Almere:**

HIV treating physicians: J. Branger*, R.A. Douma. HIV nurse consultant: A.S. Cents-Bosma, C.J.H.M. Duijf-van de Ven.

**HagaZiekenhuis, Den Haag:**

HIV treating physicians: E.F. Schippers*, C. van Nieuwkoop. HIV nurse consultants: J. Geilings, S. van Winden. HIV data collection: G. van der Hut. HIV clinical virologists/chemists: N.D. van Burgel.

**HMC (Haaglanden Medisch Centrum), Den Haag:**

HIV treating physicians: E.M.S. Leyten*, L.B.S. Gelinck, F. Mollema. HIV nurse consultants: S. Davids-Veldhuis, C. Tearno, G.S. Wildenbeest. HIV clinical virologists/chemists: E. Heikens.

**Isala, Zwolle:**

HIV treating physicians:

P.H.P. Groeneveld*, J.W. Bouwhuis, A.J.J. Lammers. HIV nurse consultants: A.G.W. van Hulzen, S. Kraan, M.S.M. Kruiper. HIV data collection: G.L. van der Bliek, P.C.J. Bor. HIV clinical virologists/chemists: S.B. Debast, G.H.J. Wagenvoort.

**Leids Universitair Medisch Centrum, Leiden:**

HIV treating physicians:

A.H.E. Roukens*, M.G.J. de Boer, H. Jolink, M.M.C. Lambregts, H. Scheper.

HIV nurse consultants: W. Dorama, N. van Holten. HIV clinical virologists/chemists:

E.C.J. Claas, E. Wessels.

**Maasstad Ziekenhuis, Rotterdam:**

HIV treating physicians: J.G. den Hollander*, R. El Moussaoui, K. Pogany. HIV nurse consultants: C.J. Brouwer, D. Heida-Peters, E. Mulder, J.V. Smit, D. Struik-Kalkman.

HIV data collection: T. van Niekerk. HIV clinical virologists/chemists: O. Pontesilli, C. van Tienen.

**Maastricht UMC+, Maastricht:**

HIV treating physicians: S.H. Lowe*, A.M.L. Oude Lashof, D. Posthouwer, M.E. van Wolfswinkel. HIV nurse consultants: R.P. Ackens, K. Burgers, M. Elasri, J. Schippers.

HIV clinical virologists/chemists: T.R.A. Havenith, M. van Loo.

**Medisch Centrum Leeuwarden, Leeuwarden:**

HIV treating physicians: M.G.A. van Vonderen*, L.M. Kampschreur. HIV nurse consultants: M.C. van Broekhuizen, S, Faber. HIV clinical virologists/chemists: A. Al Moujahid.

**Medisch Spectrum Twente, Enschede:**

HIV treating physicians: G.J. Kootstra*, C.E. Delsing. HIV nurse consultants: M. van der Burg-van de Plas, L. Scheiberlich.

**Noordwest Ziekenhuisgroep, Alkmaar:**

HIV treating physicians: W. Kortmann*, G. van Twillert*, R. Renckens, J. Wagenaar.

HIV nurse consultants & HIV data collection: D. Ruiter-Pronk, F.A. van Truijen-Oud.

HIV clinical virologists/chemists: J.W.T. Cohen Stuart, M. Hoogewerf, W. Rozemeijer, J.C. Sinnige.

**OLVG, Amsterdam:**

HIV treating physicians: K. Brinkman*, G.E.L. van den Berk, K.D. Lettinga, M. de Regt, W.E.M. Schouten, J.E. Stalenhoef, J. Veenstra, S.M.E. Vrouenraets. HIV nurse consultants: H. Blaauw, G.F. Geerders, M.J. Kleene, M. Knapen, M. Kok, I.B. van der Meché, A.J.M. Toonen, S. Wijnands, E. Wttewaal. HIV clinical virologists: D. Kwa, T.J.W. van de Laar.

**Radboudumc, Nijmegen:**

HIV treating physicians: R. van Crevel*, K. van Aerde, A.S.M. Dofferhoff, S.S.V. Henriet, H.J.M. ter Hofstede, J. Hoogerwerf, O. Richel. HIV nurse consultants: M. Albers, K.J.T. Grintjes-Huisman, M. de Haan, M. Marneef. HIV clinical virologists/chemists: M. McCall. HIV clinical pharmacology consultant: D. Burger.

**Rijnstate, Arnhem:** HIV treating physicians: E.H. Gisolf*, M. Claassen, R.J. Hassing,. HIV nurse consultants: G. ter Beest, P.H.M. van Bentum, M. Gelling, Y. Neijland. HIV clinical virologists/chemists: C.M.A. Swanink, M. Klein Velderman.

**Spaarne Gasthuis, Haarlem:**

HIV treating physicians: S.F.L. van Lelyveld*, R. Soetekouw. HIV nurse consultants: L.M.M. van der Prijt, J. van der Swaluw. HIV clinical virologists/chemists: J.S. Kalpoe, A. Wagemakers, A. Vahidnia.

**Medisch Centrum Jan van Goyen, Amsterdam:**

HIV treating physicians: F.N. Lauw, D.W.M. Verhagen. HIV nurse consultants: M. van Wijk.

**Universitair Medisch Centrum Groningen, Groningen:**

HIV treating physicians: W.F.W. Bierman*, M. Bakker, R.A. van Bentum, M.A. van den Boomgaard, J. Kleinnijenhuis, E. Kloeze, A. Middel, D.F. Postma, Y. Stienstra, M. Wouthuyzen-Bakker. HIV nurse consultants: A. Boonstra, H. de Groot-de Jonge, M.M.M. Maerman, P.A. van der Meulen, D.A. de Weerd. HIV clinical virologists/chemists: K.J. van Eije, M. Knoester, C.C. van Leer-Buter, H.G.M. Niesters.

**Beatrix Kinderziekenhuis (Universitair Medisch Centrum Groningen), Groningen:**

HIV treating physicians: E.H. Schölvinck, A.R. Verhage. HIV nurse consultants: H. de Groot-de Jonge. HIV clinical virologists/chemists: M. Knoester, C.C. van Leer-Buter, H.G.M. Niesters.

**Universitair Medisch Centrum Utrecht, Utrecht:**

HIV treating physicians: T.Mudrikova*, R.E. Barth, A.H.W. Bruns, P.M. Ellerbroek, M.P.M. Hensgens, J.J. Oosterheert, E.M. Schadd, A. Verbon, B.J. van Welzen. HIV nurse consultants: H. Berends, B.M.G. Griffioen-van Santen, I. de Kroon. HIV clinical virologists/chemists: F.M. Verduyn Lunel, A.M.J. Wensing.

**Wilhelmina Kinderziekenhuis, UMC Utrecht, Utrecht:**

HIV treating physicians: S.P.M. Geelen, Y.G.T. Loeffen, T.F.W. Wolfs. HIV nurse consultants: M. Kok. HIV clinical virologists/chemists: F.M. Verduyn Lunel, A.M.J. Wensing.

Curaçao Medical Center, Willemstad (Curaçao): HIV treating physicians: E.O.W. Rooijakkers, D. van de Wetering. HIV nurse consultants: A. Alberto. Data collection: I. der Meer.

**Coordinating center**

Board of directors: M. van der Valk, S. Zaheri.

HIV data analysis: A.C. Boyd, D.O. Bezemer, A.I. van Sighem, C. Smit, F.W.M.N. Wit

Data HIV data management and quality control: M.M.J. Hillebregt, T.J. Woudstra, T. Rutkens

HIV data monitoring: D. Bergsma, N.M. Brétin, K.J. Lelivelt, L. van de Sande, K.M. Visser

S.T. van der Vliet

HIV data collection: F. Paling, L.G.M. de Groot-Berndsen, M. van den Akker, R. Alexander, Y. Bakker, A. El Berkaoui, M. Bezemer-Goedhart, E.A. Djoechro, M. Groters, L.E. Koster, C.R.E. Lodewijk, E.G.A. Lucas, L. Munjishvili, B.M. Peeck, C.M.J. Ree, R. Regtop, A.F. van Rijk, Y.M.C. Ruijs-Tiggelman, P.P. Schnörr, M.J.C. Schoorl, E.M Tuijn, D.P. Veenenberg, E.C.M Witte.

Patiëntregistratie: Y.M.C. Ruijs-Tiggelman, D. Bergsma.
